# Socioeconomic differences in metabolic syndrome development: examining the mediating role of chronic stress using the Lifelines Cohort Study

**DOI:** 10.1186/s12889-022-12684-1

**Published:** 2022-02-08

**Authors:** Liza A. Hoveling, Aart C. Liefbroer, Ute Bültmann, Nynke Smidt

**Affiliations:** 1 Department of Epidemiology, University of Groningen, University Medical Center Groningen, PO Box 30.001, Groningen, 9700 RB The Netherlands; 2 Netherlands Interdisciplinary Demographic Institute, PO Box 11650, The Hague, 2502 AR The Netherlands; 3 Department of Sociology, Vrije Universiteit Amsterdam, De Boelelaan 1105, Amsterdam, 1081 HV The Netherlands; 4 Department of Health Sciences, Community and Occupational Medicine, University of Groningen, University Medical Center Groningen, PO Box 30.001, Groningen, 9700 RB The Netherlands

**Keywords:** Metabolic syndrome, Socioeconomic factors, Long-term difficulties inventory, Longitudinal studies, Mediation

## Abstract

**Background:**

Metabolic syndrome (MetS) development strongly varies based on individuals’ socioeconomic position (SEP), but to date, no studies have assessed the mediating role of perceived stress from long-term difficulties (chronic stress) in this association. The aim of this study is to examine the mediating role of chronic stress in the associations of the SEP measures education, occupational prestige and income, with MetS development, and whether associations between chronic stress and MetS are moderated by sex.

**Methods:**

We used an adult subsample (*n* = 53,216) from the Lifelines Cohort Study without MetS at baseline. MetS development was measured 3.9 years after baseline (follow-up), and defined according to National Cholesterol Education Program’s Adult Treatment Panel III (NCEP-ATPIII) criteria. Direct associations between SEP, chronic stress and MetS development were estimated using multivariable logistic and linear regression analyses, and were adjusted for age, sex, the other SEP measures, and time between baseline and follow-up. The mediating percentages of chronic stress explaining the associations between SEP and MetS development were estimated using the Karlson-Holm-Breen method.

**Results:**

Upon follow-up, 7.4% of the participants had developed MetS. Years of education and occupational prestige were inversely associated with MetS development. Chronic stress suppressed the association between education and MetS development (5.6%), as well as the association between occupational prestige and MetS development (6.2%). No effect modification of sex on the chronic stress-MetS pathway was observed.

**Conclusions:**

Chronic stress does not explain educational and occupational differences in developing MetS. In fact, individuals with more years of education or higher occupational prestige perceive more chronic stress than their lower SEP counterparts. Further, no difference between males and females was observed regarding the relationship between chronic stress and MetS development.

**Supplementary Information:**

The online version contains supplementary material available at 10.1186/s12889-022-12684-1.

## Introduction

Socioeconomic health differences in chronic diseases are a significant public health issue, and are expected to increase [1]. Individuals with a lower socioeconomic position (SEP) have a greater risk of developing adverse health outcomes like cardiovascular diseases (CVD) and metabolic syndrome (MetS) than their higher SEP counterparts [2, 3]. One modifiable factor that may play a role in socioeconomic health differences is the experience of stress during life [4].

Perceived stress from long-term difficulties can be defined as stress experienced over a prolonged period of time, and over which an individual perceives little or no control (referred to as ‘chronic stress’) [5]. Chronic stress is a complex, multifaceted construct, and associations with SEP may vary along multiple dimensions, including stress domain, duration, and severity [6]. Low SEP individuals are more likely to experience chronic stress from difficulties such as problems with relationships, finances, and work [7, 8]. Chronic stress is, in turn, associated with adverse health outcomes [9, 10]. Biologically, stress activates the hypothalamic-pituitary-adrenal (HPA) axis, the sympathetic nervous system, and the sympathoadrenal system, resulting in a release of cortisol and catecholamines and activation of other endocrine systems. Chronic stress and poor coping lead to disruption of the above mentioned processes as well as activation of compensatory mechanisms like allostatic load, and these reactive processes no longer adapt to ‘normal’. These factors in turn influence behavior, metabolism and immunity [11]. Direct effects of the chronic release of cortisol and catecholamines, and the activation of other endocrine systems, are: energy storage as fat, dysregulated carbohydrate metabolism, accumulation of blood lipids and increasing clotting factors, and the heart’s increased demand for oxygen while arteries are simultaneously narrowing [11, 12]. These effects may contribute to the development of MetS, a precursor of CVD [13, 14].

MetS includes at least three of the following conditions: abdominal obesity, elevated blood triglyceride levels, reduced blood high-density lipoprotein (HDL) cholesterol levels, elevated blood pressure, and elevated fasting blood glucose levels [14]. Although each of these risk factors is independently associated with increased risk of CVD, when clustered they have greater predictive value due to synergistic effects [14]. SEP and chronic stress, separately, are strongly associated with MetS development [3, 10, 15–17]. However, how and to what extent chronic stress contributes to SEP differences in the development of MetS is unknown, intervening on chronic stress could be an important step towards reducing SEP differences in MetS development.

Additionally, differences may exist between sexes in the relationship between chronic stress and MetS development, especially in those susceptible to developing an abnormal allostatic load. Despite epidemiological studies finding mixed results, different biological mechanisms for chronic stress exist between males and females [10, 18]. The above mentioned biological mechanisms could also imply derangements in the regulation of neuroendocrine and peripheral actions of sex hormones, such as androgens and estrogens. Deregulations of the HPA axis and sex hormones are thought to interact mutually in determining an abnormal response to chronic stress. For example, androgens for males and estrogens for females play different roles in fat storage. Fat storage, in turn, plays a role in the severity of obesity and metabolic alterations [18]. In addition to biological differences, sex differences exist in coping with chronic stress. Females tend to choose other coping mechanisms than males when feeling stressed, and to appraise certain situations as more stressful [19]. Given that the response between the sexes to chronic stress, and its effect on metabolic alterations, remain complex, investigating this relation could provide clues for intervening on such stress in males and females.

**Fig. 1:**
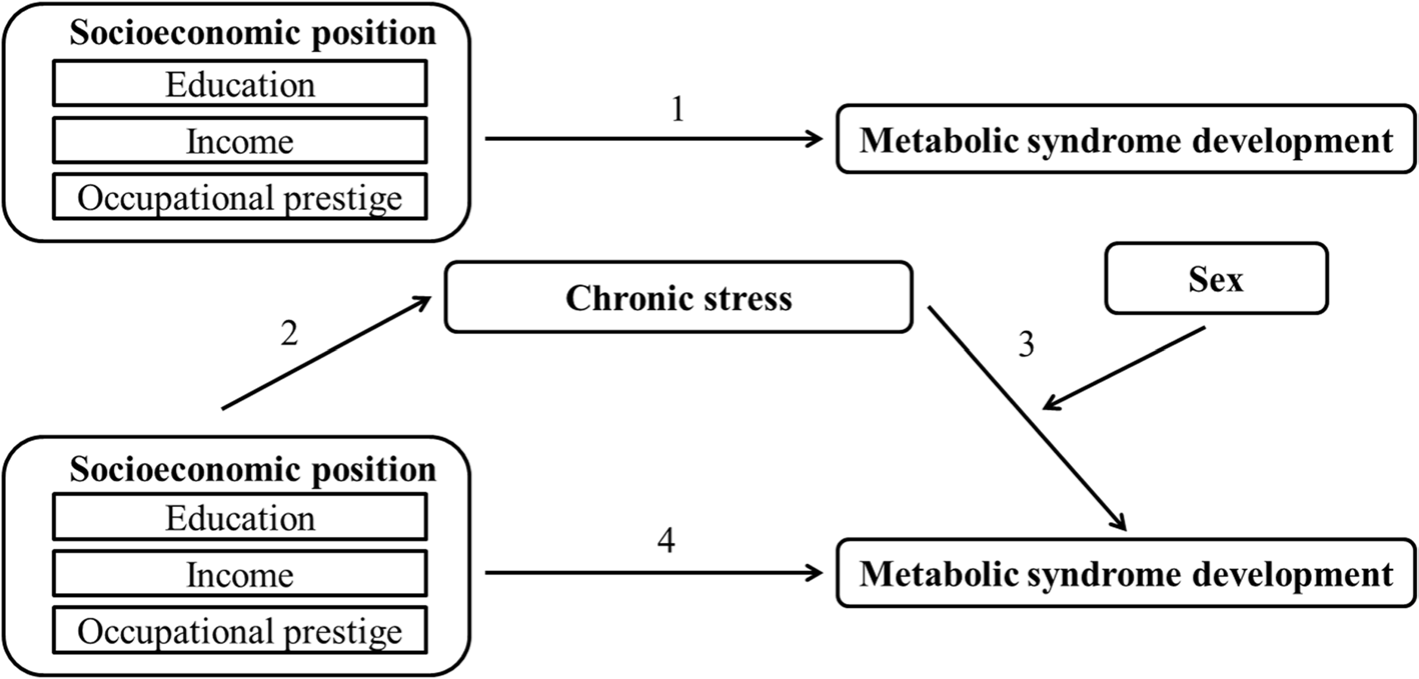
Graphical representation of direct associations between socioeconomic position and metabolic syndrome development, indirect associations via chronic stress, and effect modification of sex on the association between chronic stress and metabolic syndrome development

Despite the anticipated widening in SEP differences in health, the link between SEP and chronic stress, and the link between chronic stress and MetS development, little research has focused on how (chronic) stress affects SEP differences in health [4]. More specifically, little is known about how and to what extent chronic stress affects SEP differences in MetS development. To examine this is, therefore, our first aim (Fig. 1). We here examine three different measures of SEP: educational attainment, occupational prestige, and household income. Each of these three measures represents another important individual resource: cultural resources (education), social resources (occupational prestige), or economic resources (household income) [20, 21]. Our second aim is to investigate to what extent, if any, the association between chronic stress and MetS development is moderated by sex.

## Methods

### Study design and sample

Our study sample was derived from the Lifelines Cohort Study [22]. Lifelines is a multi-disciplinary prospective population-based cohort study, examining in a unique three-generation design the health and health-related behaviors of 167,729 persons living in the north of the Netherlands. Lifelines employs a broad range of investigative procedures to assess the biomedical, socio-demographic, behavioral, physical and psychological factors that contribute to the health and disease of the general population, with a special focus on multi-morbidity and complex genetics. The study profile of Lifelines, the recruitment, and the data collection are described elsewhere [22]. Baseline assessment (T1), consisting of a physical examination, collecting blood and urine samples, interviews and self-report questionnaires, was conducted between 2006 and 2013. Data collection was performed at baseline (T1), and on average 1.5 years (T2), 2.5 years (T3) and 3.9 years (T4) after baseline (Supplementary Fig. 1) [22].

The current study used a subsample of 120,177 participants of 18 years and older, who did not have MetS at T1, and whose data were complete for ≥70% of the variables at T1. Participants who were lost to follow-up at T4 (*n* = 29,889), for whom no MetS status could be determined based on data at T4 (*n* = 5877), or who had > 30% missing values at T1 (*n* = 31,195), were excluded from analysis (Supplementary Figure 2). Also excluded were participants with three or more MetS indicators missing, or who had provided information on only three or four indicators, so that we were unable to determine whether they had MetS [14]. Finally, 53,216 participants were included in the analyses.

### Measures and procedures

#### Socioeconomic position

SEP was defined by years of education, equivalized household income, and occupational prestige, as measured with self-reported questionnaires at T1 [22]. *Educational* level was recoded into years of education, using the number of years it would take to complete each level by the fastest route possible (see Supplementary Table 1 for measurements of the relevant variables in the Lifelines Cohort Study) [23]. *Income* was recoded as equivalized household income, determined by dividing the midpoint of each participant’s net household income category by the square root of his or her household size [24]. The amounts were divided by 100; the model estimates thus show the difference in odds ratio (OR) of MetS for a 100-euro difference in equivalized household income. *Occupational prestige* was recoded from the International Standard Classification of Occupations 2008 (ISCO08) [25] to the continuous Standard International Occupational Prestige Scale 2008 (SIOPS08) [26] and divided by 10; the model estimates thus show the difference in OR of MetS for a 10-point difference in occupational prestige score. SIOPS08 is a continuous scale, ranging from 0 to 100, and indicating low to high occupational prestige [27].

#### Metabolic syndrome

MetS indicators were measured during the physical examination and blood sample collection at T1 and T4 [22]. MetS was considered present when, according to the National Cholesterol Education Program’s Adult Treatment Panel III (NCEP-ATPIII), at least three of the five indicators were present [14]. At T2 and T3 MetS was not assessed. MetS criteria are: 1) Waist circumference ≥ 102 cm in male or ≥ 88 cm in female; 2) Systolic blood pressure ≥ 130 mmHg, diastolic blood pressure ≥ 85 mmHg, or use of blood pressure-lowering medication; 3) Triglycerides ≥150 mg/dL (1.7 mmol/l), or use of medication for elevated triglycerides; 4) HDL cholesterol < 40 mg/dL (1.0 mmol/L) in male, or < 50 mg/dL (1.3 mmol/L) in female, or use of lipid-lowering medication; 5) Fasting blood glucose level ≥ 100 mg/dL (≥ 5.6 mmol/l), diagnosis of type 2 diabetes, or use of blood glucose-lowering medication. Medication use at T1 was classified according to the Anatomical Therapeutic Chemical coding scheme [28], and at T4 with a general question about current medication use (yes/no). For every participant MetS status (yes/no) was dichotomized.

#### Long-term difficulties perceived as stressful

Long-term difficulties perceived as stressful (‘chronic stress’) were assessed using the Long-term Difficulties Inventory (LDI), a self-reported questionnaire [5, 22]. The LDI consists of 12 items evaluating to what extent various domains of life including housing, work, social relationships (relationships with friends or acquaintances, partner, children, parents or relatives), free time, finances, health, school/study, and religion had been perceived as stressful during the last year. A three-point Likert-scale was used for each item, ranging from 0 (not stressful) to 2 (very stressful). In this study, chronic stress was measured on average 1.5, 2.5 and 3.9 years after T1 (at T2, T3 and T4), after which a continuous variable indicating the total chronic stress between T1 and T4 was calculated (‘sum score’ range 0-72). In addition, chronic stress per domain of life was calculated (range 0-6) and grouped under three categories: not stressful (0), slightly stressful (1-3), and very stressful (4-6). Three domains, chronic stress: 1) at or with work, 2) with partner, and 3) with finances, are considered the most important chronic stress domains, and were displayed separately.

#### Covariates

Age and sex at T1, and time between T1 and T4, were used as control variables in all models. Covariates that may influence specific models (e.g., partner status, for chronic stress from difficulties related to partners; or work status, for chronic stress from difficulties related to work) were added to the specific models.

### Statistical analysis

Multivariable logistic- and linear regression analyses, controlling for age, sex, covariates that could influence the specific models, and other SEP measures at T1 and time between T1 and T4, were used to estimate the direct associations between SEP, chronic stress and MetS development (Fig. 1, paths 1, 2 and 3). The association between the chronic stress domains and MetS (path 3) was tested for moderation by sex by adding interaction terms between sex and chronic stress to the models. The total, direct, and indirect associations between SEP and MetS, via chronic stress and the mediating percentages of chronic stress, were estimated using the Karlson-Holm-Breen (KHB) method [29]. The KHB method was used to decompose the total effects of SEP measures on MetS development in the non-linear models into the sum of direct and indirect effects. We used the KHB method, since parameter estimates across nested non-linear models cannot be directly compared because regression coefficients and their error variance are not separately identified; this results in different error variances across models [30]. This problem of ‘rescaling’ of the error variance across nested models makes it impossible to simply examine the change in the effect of SEP on MetS development after inclusion of the chronic stress variables. The KHB method adjusts for this rescaling, and provides unbiased estimates of how much each domain-specific chronic stress variable mediates the association between the SEP measures and MetS development, depending on the presence of the other domain-specific chronic stress variables in the model [29, 30]. The results of all steps are presented as OR with 99% Confidence Intervals (CI), using ‘not stressful’ as reference category. Missing values on SEP measures and chronic stress were imputed using the Multiple Imputation by Chained Equation (MICE) method (10 imputed samples drawn every 100 iterations) [31]. To improve the quality of the imputed values, in addition to the variables used in the substantive models we added length and weight as auxiliary variables to the imputation model [32]. The imputation model included the independent variables, the mediating variables, the dependent variables, the auxiliary variables, age and sex.

By means of sensitivity analyses the robustness of the results was evaluated. To assess the potential role of misclassification of medication use at T4, analyses were repeated for a study sample only of participants who did not use medication at T4 (*n* = 31,358). To assess the potential role of selection bias from excluding participants with more than 30% missing variables, analyses were repeated with a study sample that included such participants (*n* = 85,957). Finally, a complete case analysis was performed to investigate differences in associations between the study population with imputed data and the complete cases (*n* = 41,455). In an additional analysis, the SEP-MetS relationship models were tested for moderation by sex by adding to the models interaction terms with SEP. All analyses were performed using StataMP 13 (64-bit). To allow for multiple testing, *p*-values< 0.01 were considered to be statistically significant.

## Results

The mean age of the 53,216 participants was 45.2 (SD 12.2) years, and 61.5% were female (Table 1). The majority had finished secondary vocational education, senior general secondary education, or a work-based learning pathway (39.0%); the mean net equivalized household income was 1573.8 (SD 571.3) euros per month; and the mean occupational prestige was 43.9 (SD 13.4) (e.g., dental assistant). Most participants had no chronic stress between T1 and T4 on the highlighted domains (46.5-80.2%) (see Table 2 for the chronic stress sum score and highlighted domains; see Supplementary Table 2 for all 12 domains). Correlations between the SEP measures were low to moderate (Pearson’s correlation coefficients between years of education and equivalized household income: 0.29; years of education and occupational prestige: 0.53; and equivalized household income and occupational prestige: 0.32). Overall, differences in characteristics at T1 between the study sample (*n* = 53,216) and the excluded participants (*n* = 66,961) were small (< 5%) (Supplementary Table 3). Furthermore, the differences in baseline characteristics between the study population (*n* = 53,216) and the population (*n* = 152,728) of the Lifelines Cohort Study were small (< 10%). But compared to the study population (without MetS at baseline), the population of the Lifelines Cohort Study more often perceived the 12 LDI domains as ‘very stressful’ (> 10%) (Supplementary Table 4).

**Table 1:** Baseline characteristics of the study population

Characteristics	Study population (***n*** = 53,216)^**a**^	Missing values (%)
**Demographic**		
Age (years), mean (SD)	45.2 (12.2)	0
Sex (female)	61.5	0
**Socioeconomic**		
Education (years), mean (SD)	12.3 (2.4)	2.0
Low^b^	26.5	
Middle^b^	39.0	
High^b^	32.4	
Occupational prestige (SIOPS08), mean (SD)	43.9 (13.4)	3.7
Equivalized household income (euros), mean (SD)	1573.8 (571.3)	14.5
**Metabolic syndrome indicators, meeting condition**^**c**^		
Waist circumference^d^	26.3	0
Triglyceride level^e^	8.6	0
HDL cholesterol^f^	9.1	0
Blood pressure^g^	31.6	0
Glucose level^h^	5.7	0.6
**Covariates related to Long-term Difficulties Inventory domains**		
Work (yes)	79.0	2.5
Partner (yes)	86.2	1.0
Children (yes)	73.4	2.9
Parents died (yes)	22.5	0
School/study (yes)	5.6	0
Member of a church or other religious community (yes)	22.0	0

*SD* Standard deviation, *SIOPS08* Standard International Occupational Prestige Scale 2008, *HDL* High-density lipoprotein

^a^% Presented, unless otherwise indicated

^b^Categories according to Dutch Standard Education Format [33]

^c^According to definition of metabolic syndrome by NCEP-ATPIII

^d^ ≥ 102 cm in male, or ≥ 88 cm in female

^e^ ≥ 1.70 mmol/l, or use of medication for elevated triglycerides

^f^ < 1.0 mmol/L in male, < 1.3 mmol/L in female, or use of lipid-lowering medication

^g^Systolic blood pressure ≥ 130 mmHg, diastolic blood pressure ≥ 85 mmHg, or use of blood pressure-lowering medication

^h^Fasting blood glucose level ≥ 5.6 mmol/l, diagnosis of type 2 diabetes, or use of blood glucose-lowering medication

**Table 2:** Chronic stress characteristics^a^ of the study population in questionnaires T2, T3 and T4

Chronic stress	Questionnaire T2^**b**^	Questionnaire T3^**b**^	Questionnaire T4^**b**^	Sum^**c**^
Sum score on Long-term Difficulty Inventory, mean (SD)	1.9 (2.1)	2.0 (2.2)	2.0 (2.2)	5.9 (5.6)
Missing	1.9	0.6	1.0	3.5
Work-related (e.g., too demanding, conflicts with boss, [imminent] dismissal), median (IQR)	0 (0-1)	0 (0-1)	0 (0-1)	1 (0-2)
Not stressful	65.9	68.6	63.8	46.5
Slightly stressful	27.9	25.3	28.8	44.7
Very stressful	6.1	6.1	7.2	8.5
Missing	0.1	0.0	0.2	0.3
Relationship-related (with partner) (e.g., jealousy, conflicts, doubt about the relationship, quarrels), median (IQR)	0 (0-0)	0 (0-0)	0 (0-0)	0 (0-1)
Not stressful	83.2	82.8	83.8	71.2
Slightly stressful	13.4	13.9	13.1	24.8
Very stressful	3.1	3.2	3.0	3.6
Missing	0.3	0.1	0.1	0.5
Finance-related (e.g., major debts, insufficient income), median (IQR)	0 (0-0)	0 (0-0)	0 (0-0)	0 (0-0)
Not stressful	88.7	88.0	89.9	80.2
Slightly stressful	10.0	10.6	8.9	18.1
Very stressful	1.3	1.4	1.2	1.7
Missing	0.1	0.0	0.0	0.1

*IQR* Interquartile range Q1-Q3; % presented per category ‘not stressful’ indicates sum score 0, ‘slightly stressful’ sum score 1-3, ‘very stressful’ sum score 4-6

^a^Chronic stress sum score and highlighted domains (work, partner, finances) displayed

^b^% presented, unless otherwise indicated

^c^Sum score of questionnaires T2, T3 and T4 for each Long-term Difficulties Inventory domain

### Socioeconomic position differences in metabolic syndrome development explained by chronic stress

At T4, 7.4% of the participants developed MetS. Individuals with more years of education had a lower likelihood of developing MetS (OR 0.92, 99% CI: 0.90, 0.94) (path 1) (see Table 3 for the chronic stress sum score and highlighted domains; see Supplementary Table 5 for all 12 domains). Individuals with higher occupational prestige also had a lower likelihood of developing MetS (OR 0.95, 99% CI: 0.91, 0.99). For equivalized household income, no association with MetS development was observed. Participants with higher education or higher occupational prestige were more likely to perceive chronic stress (β = 0.23, 99% CI: 0.20, 0.26 and β = 0.16, 99% CI: 0.11, 0.22) (path 2). Compared to the lower educated, participants with higher education more often perceived chronic stress related to work (slightly stressful OR 1.14, 99% CI: 1.12, 1.15, and very stressful OR 1.21, 99% CI: 1.18, 1.24). They also more often perceived chronic stress concerning their relationship with their partner (slightly stressful OR 1.06, 99% CI: 1.04, 1.08, and very stressful OR 1.12, 99% CI: 1.08, 1.15). Furthermore, the more highly educated also more often perceived chronic stress related to finances (slightly stressful OR 1.05, 99% CI: 1.03, 1.07, and very stressful OR 1.05, 99% CI: 1.00, 1.10). Participants with higher occupational prestige perceived more chronic stress related to work (slightly stressful OR 1.09, 99% CI: 1.07, 1.12, and very stressful OR 1.17, 99% CI: 1.12, 1.21) or within their relationship (slightly stressful OR 1.03, 99% CI: 1.01, 1.06, and very stressful OR 1.04, 99% CI: 0.99, 1.10) than participants with lower occupational prestige. Further, participants who perceived more chronic stress had a greater risk of developing MetS (OR 1.02, 99% CI: 1.01, 1.03) (path 3). In particular, chronic stress concerning finances increased the risk of developing MetS (slightly stressful OR 1.33, 99% CI: 1.18, 1.50, and very stressful OR 1.58, 99% CI: 1.16, 2.15).

**Table 3:** Multivariable logistic and linear regression analysis of direct associations between socioeconomic position, chronic stress^a^, and metabolic syndrome development (*n* = 53,216)

	Education	Occupational prestige	Income
	OR (99% CI)	OR (99% CI)	OR (99% CI)
**Path 1. SEP and MetS development**	0.92 (0.90, 0.94)*	0.95 (0.91, 0.99)*	0.99 (0.98, 1.00)
**Path 2. SEP and chronic stress**^**a**^			
Sum score (beta)	0.23 (0.20, 0.26)*	0.16 (0.11, 0.22)*	−0.10 (−0.11, −0.08)*
Work-related			
Slightly stressful	1.14 (1.12, 1.15)*	1.09 (1.07, 1.12)*	1.01 (1.00, 1.01)*
Very stressful	1.21 (1.18, 1.24)*	1.17 (1.12, 1.21)*	1.02 (1.01, 1.03)*
Relationship-related (with partner)			
Slightly stressful	1.06 (1.04, 1.08)*	1.03 (1.01, 1.06)*	0.98 (0.98, 0.99)*
Very stressful	1.12 (1.08, 1.15)*	1.04 (0.99, 1.10)	0.97 (0.95, 0.98)*
Finance-related			
Slightly stressful	1.05 (1.03, 1.07)*	1.00 (0.97, 1.03)	0.93 (0.92, 0.94)*
Very stressful	1.05 (1.00, 1.10)	1.00 (0.93, 1.09)	0.88 (0.86, 0.90)*
**Path 3. Chronic stress**^**a **^**and MetS development**			
Sum score	1.02 (1.01, 1.03)*	1.02 (1.01, 1.03)*	1.02 (1.01, 1.03)*
Work-related			
Slightly stressful	0.99 (0.89, 1.10)	0.99 (0.89, 1.10)	0.99 (0.89, 1.10)
Very stressful	1.07 (0.89, 1.28)	1.07 (0.89, 1.28)	1.07 (0.89, 1.28)
Relationship-related (with partner)			
Slightly stressful	0.82 (0.73, 0.92)*	0.82 (0.73, 0.92)*	0.82 (0.73, 0.92)*
Very stressful	0.80 (0.62, 1.05)	0.80 (0.62, 1.05)	0.80 (0.62, 1.05)
Finance-related			
Slightly stressful	1.33 (1.18, 1.50)*	1.33 (1.18, 1.50)*	1.33 (1.18, 1.50)*
Very stressful	1.58 (1.16, 2.15)*	1.58 (1.16, 2.15)*	1.58 (1.16, 2.15)*
**Path 4. SEP and MetS development**^**b**^			
Sum score	0.92 (0.90, 0.94)*	0.95 (0.91, 0.99)*	1.00 (0.99, 1.01)
Work-related	0.92 (0.90, 0.94)*	0.95 (0.91, 0.99)*	0.99 (0.98, 1.00)
Relationship-related (with partner)	0.92 (0.90, 0.94)*	0.95 (0.91, 0.99)*	0.99 (0.98, 1.00)
Finance-related	0.92 (0.90, 0.94)*	0.95 (0.91, 0.99)*	1.00 (0.99, 1.01)

*OR* Odds ratio, *CI* Confidence interval, *SEP* Socioeconomic position, *MetS* Metabolic syndrome, *LDI* Long-term Difficulties Inventory; analyses controlled for years of education, equivalized household income, occupational prestige, age and sex at T1, and time between T1 and T4; reference category for the LDI domains was ‘not stressful’; LDI domains were controlled for work status, partner status, children status, parent status, school/study status and religion status where applicable

*P < 0.01

^a^Long-term difficulties during total follow-up time measured with the LDI, LDI categories consist of the sum score of the LDI from questionnaires T2, T3 and T4, ‘not stressful’ indicates sum score 0, ‘slightly stressful’ sum score 1-3, ‘very stressful’ sum score 4-6

^b^Direct associations between SEP measures and MetS development controlled for specific LDI domain

Educational- and occupational differences in developing MetS were, respectively, for 5.6 and 6.2% suppressed by the chronic stress sum score (see Table 4 for the chronic stress sum score and highlighted domains; see Supplementary Table 6 for all 12 domains).

**Table 4:** Multivariable mediation analysis of chronic stress^a^ in associations between socioeconomic position and metabolic syndrome development, using the Karlson-Holm-Breen method (*n* = 53,216)

	**Education**	**Occupational prestige**	**Income**
	**OR (99% CI)**	**OR (99% CI)**	**OR (99% CI)**
**Total association**	0.92 (0.90, 0.94)*	0.95 (0.91, 0.99)*	0.99 (0.98, 1.00)
**Direct association**	0.92 (0.90, 0.94)*	0.95 (0.91, 0.99)*	1.00 (0.99, 1.00)
**Indirect association**	1.00 (1.00, 1.01)*	1.00 (1.00, 1.00)*	1.00 (1.00, 1.00)
	**Percentage**	**Percentage**	**Percentage**
**Mediating effect**			
Sum score	−5.6	−6.2	25.1
**Mediating effects per highlighted domain of life**			
Work-related	−0.5	−1.0	−1.1
Relationship-related (with partner)	2.7	2.9	−12.2
Finance-related	−2.3	0.4	51.5

*OR* Odds ratio, *CI* Confidence interval, *SEP* Socioeconomic position, *MetS* Metabolic syndrome, *LDI* Long-term Difficulties Inventory; analyses controlled for years of education, equivalized household income, occupational prestige, age and sex at T1, and time between T1 and T4; reference category for the LDI domains was ‘not stressful’; LDI domains were controlled for work status, partner status, children status, parent status, school/study status and religion status where applicable

*P < 0.01

^a^Long-term difficulties during total follow-up time measured with the LDI

### The moderating role of sex on the association between chronic stress and metabolic syndrome development

No significant interactions between the sum of the chronic stress domains, or the chronic stress domains separately, and sex on MetS development were observed (*p*-values ≥0.01 for interactions) (see Table 5 for the chronic stress sum score and highlighted domains; see Supplementary Table 7 for all 12 domains).

**Table 5:** Interaction coefficients of sex*chronic stress in multivariable logistic regression analyses between chronic stress^a^ and metabolic syndrome development (*n* = 53,216)

Chronic stress^**a**^ domain	OR (99% CI)
Female*Sum score	0.99 (0.98, 1.01)
Work-related	
Female*Not stressful	1.00
Female*Slightly stressful	0.89 (0.74, 1.07)
Female*Very stressful	0.93 (0.67, 1.30)
Relationship-related (with partner)	
Female*Not stressful	1.00
Female*Slightly stressful	0.89 (0.71, 1.10)
Female*Very stressful	0.90 (0.53, 1.52)
Finance-related	
Female*Not stressful	1.00
Female*Slightly stressful	0.89 (0.72, 1.11)
Female*Very stressful	1.26 (0.68, 2.34)

*OR* Odds ratio, *CI* Confidence interval, *LDI* Long-term Difficulties Inventory; analyses controlled for years of education, equivalized household income, occupational prestige, age and sex at T1, and time between T1 and T4; reference category for the LDI domains was ‘not stressful’; LDI domains were controlled for work status, partner status, children status, parent status, school/study status and religion status where applicable.

*P < 0.01

^a^Long-term difficulties during total follow-up time measured with the LDI, LDI categories consist of the sum score of the LDI from questionnaires T2, T3 and T4, ‘not stressful’ indicates sum score 0, ‘slightly stressful’ sum score 1-3, ‘very stressful’ sum score 4-6

Sensitivity analyses did not show results substantively different than those of the main analysis when using a study sample including only participants who used no medication at T4, a less conservative population selection, or a study sample including only complete cases (Supplementary Tables 8, 9, 10, 11, 12 and 13). Additional analysis to test the moderating effect of sex on the relationship between SEP and MetS, showed no sex differences for this relationship (*p*-values ≥0.01 for interactions) (Supplementary Table 14).

## Discussion

In line with previous work, our results show education and occupational prestige, but not equivalized household income, to be strong determinants of MetS development [3]. This indicates that financial resources (e.g., income) are less important in developing MetS than cultural and social resources (e.g., education and occupational prestige). The foregoing could have to do with the Dutch context of our study, as in the Netherlands access to health care does not depend strongly on income; knowledge and social skills seem to be more important aspects. To examine how much the role of income in developing MetS depends on the social context, it would be worthwhile to compare the results of the current study with results in social contexts other than the Netherlands, where income plays a greater role in access to health care.

Chronic stress does not explain the observed educational and occupational differences in developing MetS. In fact, individuals with more years of education or higher occupational prestige perceive more chronic stress than do their lower SEP counterparts. It so happens that chronic stress suppresses the relationship between SEP and MetS development. If individuals with a higher SEP were to experience the same levels of chronic stress as individuals with a lower SEP, the resulting SEP differences in MetS development would be even greater than those observed in our sample. In general, no indications were found that sex modifies the effect of the association between chronic stress and MetS development.

Although evidence is scarce, a review suggests that SEP differences in health may be explained by (chronic) stress [4]. However, the current study has found no indications for this. Overall, our results show positive associations between SEP and chronic stress. Higher educated participants perceive more chronic stress in general, and this pattern is also observed for the LDI domains work, partner relationship, and finances. Likewise, participants with higher occupational prestige perceive more chronic stress in general, with a similar pattern for the domains work and partner relationship. On the other hand, participants with a higher equivalized household income perceive less chronic stress; this is in line with a systematic review of SEP in relation to allostatic load [34], and this pattern is also observed for the domains partner relationship and finances. However, the positive associations we found between SEP and chronic stress are inconsistent with previous social and biological research [7, 8, 34], which in general suggests that SEP may buffer the effect of stress because of the enhanced social support, better coping style, and greater optimism of higher SEP individuals [35]. A number of factors may contribute to this inconsistency. The measures of perceived stress used in previous research are heterogeneous. Some studies have used the presence of each individual domain of perceived stress, others the sum of stress experienced, and yet others have grouped the perceived stress domains into different thematic categories [6]. Moreover, it is difficult to capture the biological concept ‘chronic stress’ in epidemiological studies such as the current study. To our knowledge, we are the first to use the broad composite LDI instrument for measuring perceived stress resulting from long-term difficulties (‘chronic stress’) in relation to SEP and MetS development. Although the LDI has been found to possess good validity and stability for use in large epidemiological studies, nevertheless, because it remains a 12 domain questionnaire with questions only up to a year ago, the occurrence of recall bias cannot be ruled out [5]. Another explanation for the inconsistency between our study and previous research could be that different SEP groups perceive and report ‘chronic stress’ differently in self-reported questionnaires; for example, due to repeated exposure to socioeconomic disadvantage, individuals with low SEP may over time become used to chronic stress. The foregoing observation is in line with the resilience theory, which argues that it is not the nature of adversity that is most important, but how the individual deals with it [36]. Individuals with a low SEP may be more resilient (e.g., due to disadvantages during childhood) to chronic stress from finances, their partner, or their work than their high SEP counterparts. Yet another explanation for the observed positive associations between SEP and chronic stress in our study could be that high SEP ensures that an individual has better cognitive skills [37], implying a more challenging job, higher demands [8], less leisure time (e.g., jointly with their partner) and a greater sense of responsibility (e.g., for their job and their partner), leading to more chronic stress. Furthermore, belonging to a social network with only high SEP individuals can make people want to belong. Certain roles may then be taken on (e.g., managerial), and if difficulties with finances or work subsequently arise, they can cause extra stress, because the person wants to remain part of the high SEP group. Although our study does not indicate whether MetS was present during every follow-up period when chronic stress was measured, consistent with previous work our results clearly show that chronic stress, and especially chronic stress related to finances, increases the risk of developing MetS [10].

The current study found no sex differences on the relationship between chronic stress and MetS development. Although between males and females different biological and social processes may play a role in the effect of chronic stress on MetS development [18, 19], other epidemiological studies have found mixed results [10]. In our study we attempted to measure ‘chronic stress’ by means of the LDI questionnaire. Even though we observed no differences between the sexes in the relationship between chronic stress and MetS development, and according to our study the associations would be the same for males and females, one cannot on the basis of our study entirely rule out a sex difference. It remains possible that (sex differences in the) biological processes that take place during chronic stress are not reflected in the self-reported LDI questionnaire.

This study has several important strengths. First, our results extend previous work, using a large representative sample and longitudinal design to assess whether chronic stress is related to SEP differences in the development of MetS. Second, experiencing stress from long-term difficulties was measured using a validated questionnaire, with 12 questions referring to different aspects of life [5]. Third, our results are likely to be generalizable to individuals without MetS in the north of the Netherlands [38]. Fourth, MetS indicators were measured during physical examinations and by drawing blood samples, thereby minimizing the risk of measurement bias in the outcome measure. A limitation of our study is that the presence of MetS at T4 was determined without accounting for specific medication use. However, the sensitivity analysis that included only participants who used no prescribed medication at T4 suggested that the associations would not differ if specific medication use was taken into account. A second limitation is that the LDI does not cover the complete follow-up period, as it asks for long-term difficulties only in the past year. However, having used a sum score of three measurements over a time-period of less than 4 years, we do not think this will have affected the results significantly. A third limitation is that we cannot rule out residual confounding; while the aim of this study was to investigate only the role of chronic stress in relation to SEP differences in MetS development, further studies should investigate other factors that may explain these differences. A fourth limitation is that, because SEP and chronic stress were measured using self-reported questionnaires, measurement bias may have occurred. However, as we anticipate that this bias will be random and not systematic in one direction, it is not expected to affect our results.

Our findings may have important implications for researchers, policymakers and healthcare professionals. Researchers should be aware that SEP differences in MetS development are not resolved by removing chronic stress. Further research should focus on other factors that may influence differences between high and low SEP in developing MetS, such as social- or environmental factors, or the interplay among factors. Moreover, as an individual's education, occupational prestige and income are related differently to experiencing chronic stress and MetS development, these three SEP measures should be considered separately. Furthermore, additional epidemiological research is needed into the effect of sex differences on the relationship between chronic stress and MetS development. Although the current study found no such sex differences, prior biological studies have supported such expectations [18]. For these reasons we recommend more epidemiological research into stress measures, so that the social aspects (e.g., features of the home environment, residential density, crowding, inadequate housing, poor sanitation, noise, fear of crime) and biological aspects of chronic stress can be better apprehended than by using a self-reported questionnaire with only 12 domains of life. Policymakers should understand that intervening on chronic stress alone is not likely to reduce SEP differences in developing MetS. Of interest for healthcare professionals is that individuals with a high SEP are more likely to suffer from chronic stress than individuals with a low SEP, and that chronic stress increases the risk of developing MetS. Also of interest is that our study suggests that the effect of chronic stress on the development of MetS is the same for males and females. 

## Conclusion

The current study indicates that educational and occupational differences in MetS development are not explained by chronic stress. Further, no difference between males and females on the relationship between chronic stress and MetS development was observed. According to our study; 1) treatments and interventions aimed at reducing chronic stress in low SEP individuals would not reduce SEP differences in the development of MetS, and 2) treatments and interventions to reduce chronic stress in order to prevent MetS development need not differ for males and females.

## Supplementary Information

**Additional file 1: Supplementary Figure 1.** Graphical representation of measurements of the key variables in the study. **Supplementary Figure 2.** Flowchart of the selection of the study population. **Supplementary Table 1.** Measurements in the Lifelines Cohort Study of the variables used in the analyses. **Supplementary Table 2.** Chronic stress characteristics of the study population in questionnaires T2, T3 and T4. **Supplementary Table 3.** Baseline characteristics of the baseline population (*n* = 120,177) and a comparison of the study population (*n* = 53,216) and the participants excluded (*n* = 66,961). **Supplementary Table 4.** Baseline characteristics of the Lifelines Cohort Study (*n* = 152,728) and a comparison of the study population (*n* = 53,216) and the participants excluded (*n* = 99,512). **Supplementary Table 5.** Multivariable logistic- and linear regression analysis of direct associations between socioeconomic position, chronic stress, and metabolic syndrome development in the study population (*n* = 53,216). **Supplementary Table 6.** Multivariable mediation analysis of chronic stress^a^ in associations between socioeconomic position and metabolic syndrome development, using the Karlson-Holm-Breen method in the study population (*n* = 53,216). **Supplementary Table 7.** Interaction coefficients of sex*chronic stress in the multivariable logistic regression analysis between chronic stress and metabolic syndrome development (*n* = 53,216). **Supplementary Table 8.** Multivariable logistic- and linear regression analysis of direct associations between socioeconomic position, chronic stress, and metabolic syndrome development among participants who did not use medication at T4 (*n* = 31,358). **Supplementary Table 9.** Multivariable mediation analysis of chronic stress in associations between socioeconomic position and metabolic syndrome development, using the Karlson-Holm-Breen method among participants who did not use medication at T4 (*n* = 31,358). **Supplementary Table 10.** Multivariable logistic- and linear regression analysis of direct associations between socioeconomic position, chronic stress, and metabolic syndrome development among participants with more than 30% missings on variables (*n* = 85,957). **Supplementary Table 11.** Multivariable mediation analysis of chronic stress in associations between socioeconomic position and metabolic syndrome development, using the Karlson-Holm-Breen method among participants with more than 30% missings on variables (*n* = 85,957). **Supplementary Table 12.** Multivariable logistic- and linear regression analysis of direct associations between socioeconomic position, chronic stress^a^, and metabolic syndrome development among complete cases (*n* = 41,455). **Supplementary Table 13.** Multivariable mediation analysis of chronic stress in associations between socioeconomic position and metabolic syndrome development, using the Karlson-Holm-Breen method among complete cases (*n* = 41,455). **Supplementary Table 14.** Interaction coefficients of sex*SEP in the multivariable logistic regression analyses between socioeconomic position measures and metabolic syndrome development in the study population (*n* = 53,216).

## Data Availability

The data that support the findings of this study are available from the Lifelines Cohort Study. Restrictions apply to their use: as they were used under license for the current study, they are not publicly available. However, data may be made available by the authors upon reasonable request and with permission of the Lifelines Cohort Study (www.lifelines.nl).
