# Supplementary material for: Socioeconomic differences in metabolic syndrome development: examining the mediating role of chronic stress using the Lifelines Cohort Study
Source: BMC Public Health. 2022 Feb 8;22:261. doi: 10.1186/s12889-022-12684-1 (PMC8827257; doi:10.1186/s12889-022-12684-1)
Supplement: Supplementary file 1 — Additional file 1: Supplementary Figure 1. Graphical representation of measurements of the key variables in the study. Supplementary Figure 2. Flowchart of the selection of the study population. Supplementary Table 1. Measurements in the Lifelines Cohort Study of the variables used in the analyses. Supplementary Table 2. Chronic stress characteristics of the study population in questionnaires T2, T3 and T4. Supplementary Table 3. Baseline characteristics of the baseline population (n = 120,177) and a comparison of the study population (n = 53,216) and the participants excluded (n = 66,961). Supplementary Table 4. Baseline characteristics of the Lifelines Cohort Study (n = 152,728) and a comparison of the study population (n = 53,216) and the participants excluded (n = 99,512). Supplementary Table 5. Multivariable logistic- and linear regression analysis of direct associations between socioeconomic position, chronic stress, and metabolic syndrome development in the study population (n = 53,216). Supplementary Table 6. Multivariable mediation analysis of chronic stressa in associations between socioeconomic position and metabolic syndrome development, using the Karlson-Holm-Breen method in the study population (n = 53,216). Supplementary Table 7. Interaction coefficients of sex*chronic stress in the multivariable logistic regression analysis between chronic stress and metabolic syndrome development (n = 53,216). Supplementary Table 8. Multivariable logistic- and linear regression analysis of direct associations between socioeconomic position, chronic stress, and metabolic syndrome development among participants who did not use medication at T4 (n = 31,358). Supplementary Table 9. Multivariable mediation analysis of chronic stress in associations between socioeconomic position and metabolic syndrome development, using the Karlson-Holm-Breen method among participants who did not use medication at T4 (n = 31,358). Supplementary Table 10. Multivariable logistic- and linea [file 12889_2022_12684_MOESM1_ESM.docx]

**SUPPLEMENTARY MATERIAL**


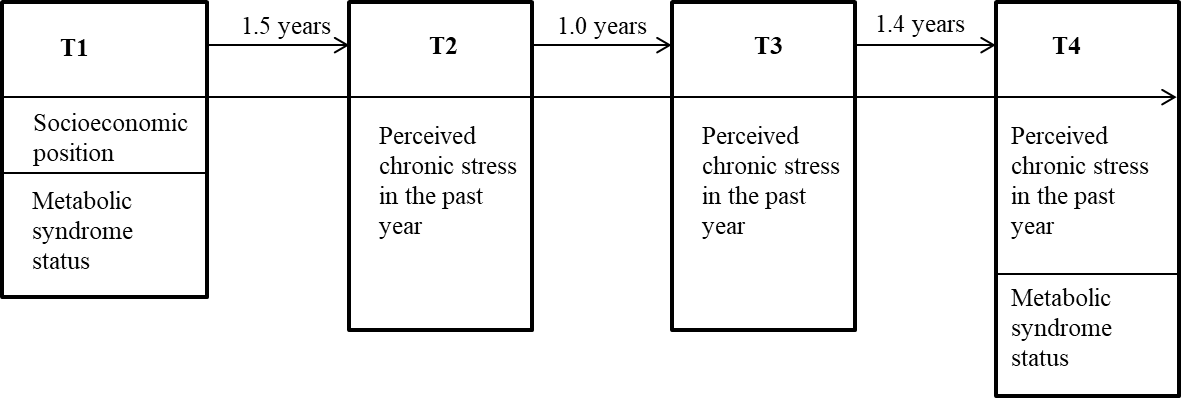


**Supplementary Figure 1. Graphical representation of measurements of the key variables in the study.**

**
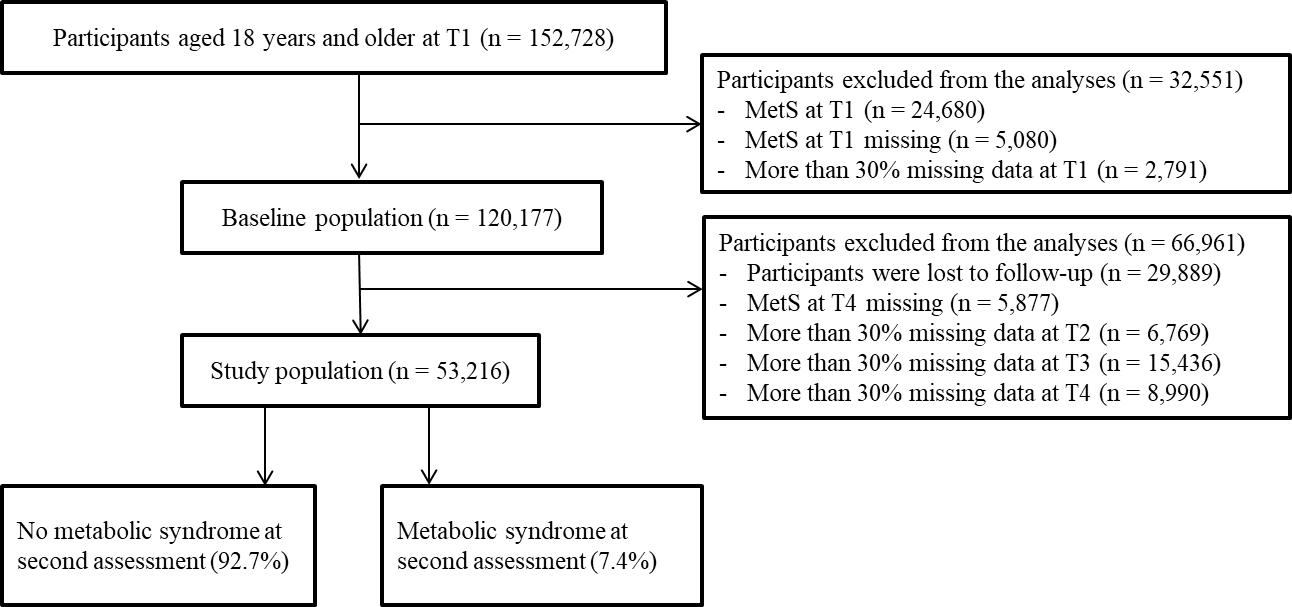
**

**Supplementary Figure 2. Flowchart of the selection of the study population.**

**Supplementary Table 1. Measurements in the Lifelines Cohort Study of the variables used in the analyses.**

| **Variables** | **Measured in the Lifelines Cohort Study** |
| --- | --- |
| Education | ‘What is the highest level of education you have completed?’ Participants had eight answer options, including ‘No education’, ‘Primary education’, ‘Lower or preparatory secondary vocational education’, ‘Junior general secondary education’, ‘Secondary vocational education or work-based learning pathway’, ‘Senior general secondary education, pre-university secondary education’, ‘Higher vocational education’ and ‘University education’. |
| Equivalized household income | Income was measured with the question ‘What is the net income per month. So what you receive in cash and/or on your bank/giro. PLEASE NOTE: if you share the household with someone, the income of your partner(s) must also be included.’. Participants had ten answer options, including ‘Less than 750 euros’, ‘750-1000 euros’, ‘1000-1500 euros’, ‘1500-2000 euros’, ‘2000-2500 euros’, ‘2500-3000 euros’, ‘3000-3500 euros’, ‘more than 3500 euros’, ‘I do not know this.’, ‘I prefer not to answer that.’. |
|  | Household size was measured with the question ‘How many people live from this income?’ ranging from 1 to more than 6 persons. |
| Occupational prestige | ‘If you have or had paid work, what is your last or current profession?’ and ‘Can you explain your profession or function by describing what your main activities are or were?’. Answers were categorized according to the International Standard Classification of Occupations 2008 (ISCO08). |
| Waist circumference | During the physical examination using the SECA 200 measuring tape. |
| Blood pressure | Measurements at T1 consisted of 10 measurements during 10 minutes using the Dinamap PRO 100V2. During T4 measurements consisted of three measurements using the Dinamap PRO 100V2. At both occasions, blood pressure was calculated as the average of the last two measurements. |
| Triglyceride | Measured during a laboratory assessment on the day of the fasten blood sample collection. |
| HDL cholesterol | Measured during a laboratory assessment on the day of the fasten blood sample collection. |
| Fasting blood glucose | Measured during a laboratory assessment on the day of the fasten blood sample collection. If participants indicated they did not fast before the blood sample collection, their blood glucose values were assumed not to be valid if they exceeded 5.6 mmol/L according to the cut-off for having MetS and were interpreted as missing value. The determination of type 2 diabetes was based on self-reported questionnaires. |
| Medication use at T1 | The participant was asked about medication use through a questionnaire and by bringing used medications to the research site upon time of the physical examination. All prescribed medications were classified according to the Anatomical Therapeutic Chemical (ATC) coding scheme [28]. |
| Medication use at T4 | ‘Do you use prescribed medication?’, participants answering yes or no. Specific information about the prescribed medication was not available at T4. Measurement of these components in the Lifelines Cohort Study was performed in the same way at T1 and T4 unless otherwise indicated. |
| Long-term difficulties related to: | |
| Home and living (e.g., accommodation too small, could not find a home, noise) | ‘To what extent did you experience difficulties and stress related to this aspect of your life?’ Participants could answer on a three point Likert-scale, ranging from 0 (not stressful) to 2 (very stressful). |
| At or with work (e.g., too demanding, conflicts with boss, (imminent) dismissal) | ‘To what extent did you experience difficulties and stress related to this aspect of your life?’ Participants could answer on a three point Likert-scale, ranging from 0 (not stressful) to 2 (very stressful). |
| Relationship with friends or acquaintances (e.g., quarrels, lack of support) | ‘To what extent did you experience difficulties and stress related to this aspect of your life?’ Participants could answer on a three point Likert-scale, ranging from 0 (not stressful) to 2 (very stressful). |
| Relationship with your partner (e.g., jealousy, conflicts, doubt about the relationship, quarrels) | ‘To what extent did you experience difficulties and stress related to this aspect of your life?’ Participants could answer on a three point Likert-scale, ranging from 0 (not stressful) to 2 (very stressful). |
| Relationship with your children (e.g., frequent conflicts, lack of respect for you) | ‘To what extent did you experience difficulties and stress related to this aspect of your life?’ Participants could answer on a three point Likert-scale, ranging from 0 (not stressful) to 2 (very stressful). |
| Relationship with your parents (e.g., frequent conflicts, lack of acceptance) | ‘To what extent did you experience difficulties and stress related to this aspect of your life?’ Participants could answer on a three point Likert-scale, ranging from 0 (not stressful) to 2 (very stressful). |
| Relationship with other relatives (e.g., conflicts, lack of acceptance) | ‘To what extent did you experience difficulties and stress related to this aspect of your life?’ Participants could answer on a three point Likert-scale, ranging from 0 (not stressful) to 2 (very stressful). |
| Free time (e.g., too little or too much free time) | ‘To what extent did you experience difficulties and stress related to this aspect of your life?’ Participants could answer on a three point Likert-scale, ranging from 0 (not stressful) to 2 (very stressful). |
| Finances (e.g., major debts, insufficient income) | ‘To what extent did you experience difficulties and stress related to this aspect of your life?’ Participants could answer on a three point Likert-scale, ranging from 0 (not stressful) to 2 (very stressful). |
| Your health (e.g., regularly ill, longer-term disorders) | ‘To what extent did you experience difficulties and stress related to this aspect of your life?’ Participants could answer on a three point Likert-scale, ranging from 0 (not stressful) to 2 (very stressful). |
| School/study (too difficult, cannot be combined with other tasks) | ‘To what extent did you experience difficulties and stress related to this aspect of your life?’ Participants could answer on a three point Likert-scale, ranging from 0 (not stressful) to 2 (very stressful). |
| Faith, church or religion (e.g., doubt, conflicts with your minister) | ‘To what extent did you experience difficulties and stress related to this aspect of your life?’ Participants could answer on a three point Likert-scale, ranging from 0 (not stressful) to 2 (very stressful). |

**Supplementary Table 2. Chronic stress characteristics^a^ of the study population in questionnaires T2, T3 and T4.**

| **Chronic stress** | **Questionnaire T2^b^** | **Questionnaire T3^b^** | **Questionnaire T4^b^** | **Sum^c^** |
| --- | --- | --- | --- | --- |
| Sum score on Long-term Difficulty Inventory, mean (SD) | 1.9 (2.1) | 2.0 (2.2) | 2.0 (2.2) | 5.9 (5.6) |
| Missing | 1.9 | 0.6 | 1.0 | 3.5 |
| Home and living (e.g., accommodation too small, could not find a home, noise), median (IQR) | 0 (0-0) | 0 (0-0) | 0 (0-0) | 0 (0-0) |
| Not stressful | 90.1 | 89.8 | 89.1 | 79.7 |
| Slightly stressful | 8.4 | 8.6 | 9.2 | 18.7 |
| Very stressful | 1.4 | 1.6 | 1.7 | 1.5 |
| Missing | 0.1 | 0.0 | 0.0 | 0.1 |
| At or with work (e.g., too demanding, conflicts with boss, [imminent] dismissal), median (IQR) | 0 (0-1) | 0 (0-1) | 0 (0-1) | 1 (0-2) |
| Not stressful | 65.9 | 68.6 | 63.8 | 46.5 |
| Slightly stressful | 27.9 | 25.3 | 28.8 | 44.7 |
| Very stressful | 6.1 | 6.1 | 7.2 | 8.5 |
| Missing | 0.1 | 0.0 | 0.2 | 0.3 |
| Relationship with friends or acquaintances (e.g., quarrels, lack of support), median (IQR) | 0 (0-0) | 0 (0-0) | 0 (0-0) | 0 (0-0) |
| Not stressful | 89.8 | 89.1 | 90.2 | 78.5 |
| Slightly stressful | 9.2 | 10.0 | 8.9 | 20.7 |
| Very stressful | 0.9 | 0.9 | 0.8 | 0.7 |
| Missing | 0.1 | 0.0 | 0.1 | 0.2 |
| Relationship with your partner (e.g., jealousy, conflicts, doubt about the relationship, quarrels) , median (IQR) | 0 (0-0) | 0 (0-0) | 0 (0-0) | 0 (0-1) |
| Not stressful | 83.2 | 82.8 | 83.8 | 71.2 |
| Slightly stressful | 13.4 | 13.9 | 13.1 | 24.8 |
| Very stressful | 3.1 | 3.2 | 3.0 | 3.6 |
| Missing | 0.3 | 0.1 | 0.1 | 0.5 |
| Relationship with your children (e.g., frequent conflicts, lack of respect for you) , median (IQR) | 0 (0-0) | 0 (0-0) | 0 (0-0) | 0 (0-0) |
| Not stressful | 85.5 | 85.4 | 86.2 | 75.3 |
| Slightly stressful | 12.7 | 13.0 | 12.3 | 22.1 |
| Very stressful | 1.3 | 1.4 | 1.4 | 1.8 |
| Missing | 0.6 | 0.1 | 0.2 | 0.9 |
| Relationship with your parents (e.g., frequent conflicts, lack of acceptance) , median (IQR) | 0 (0-0) | 0 (0-0) | 0 (0-0) | 0 (0-0) |
| Not stressful | 90.2 | 90.4 | 90.8 | 82.1 |
| Slightly stressful | 7.9 | 8.2 | 7.6 | 15.3 |
| Very stressful | 1.2 | 1.2 | 1.2 | 1.4 |
| Missing | 0.7 | 0.3 | 0.4 | 1.4 |
| Relationship with other relatives (e.g., conflicts, lack of acceptance) , median (IQR) | 0 (0-0) | 0 (0-0) | 0 (0-0) | 0 (0-0) |
| Not stressful | 87.4 | 87.0 | 87.6 | 75.0 |
| Slightly stressful | 11.1 | 11.7 | 11.1 | 23.5 |
| Very stressful | 1.4 | 1.3 | 1.3 | 1.3 |
| Missing | 0.1 | 0.0 | 0.1 | 0.2 |
| Free time (e.g., too little or too much free time) , median (IQR) | 0 (0-1) | 0 (0-1) | 0 (0-1) | 0 (0-2) |
| Not stressful | 71.7 | 70.1 | 70.2 | 50.9 |
| Slightly stressful | 26.1 | 27.4 | 27.1 | 45.6 |
| Very stressful | 2.1 | 2.4 | 2.6 | 3.4 |
| Missing | 0.1 | 0.0 | 0.1 | 0.2 |
| Finances (e.g., major debts, insufficient income) , median (IQR) | 0 (0-0) | 0 (0-0) | 0 (0-0) | 0 (0-0) |
| Not stressful | 88.7 | 88.0 | 89.9 | 80.2 |
| Slightly stressful | 10.0 | 10.6 | 8.9 | 18.1 |
| Very stressful | 1.3 | 1.4 | 1.2 | 1.7 |
| Missing | 0.1 | 0.0 | 0.0 | 0.1 |
| Your health (e.g., regularly ill, longer-term disorders) , median (IQR) | 0 (0-0) | 0 (0-0) | 0 (0-0) | 0 (0-1) |
| Not stressful | 82.4 | 82.8 | 79.9 | 66.9 |
| Slightly stressful | 14.8 | 14.6 | 16.9 | 29.6 |
| Very stressful | 2.7 | 2.6 | 3.1 | 3.3 |
| Missing | 0.1 | 0.0 | 0.1 | 0.2 |
| School/study (too difficult, cannot be combined with other tasks) , median (IQR) | 0 (0-0) | 0 (0-0) | 0 (0-0) | 0 (0-0) |
| Not stressful | 93.5 | 94.0 | 94.3 | 87.9 |
| Slightly stressful | 5.4 | 4.9 | 4.5 | 10.5 |
| Very stressful | 0.9 | 1.0 | 1.0 | 0.9 |
| Missing | 0.3 | 0.2 | 0.3 | 0.8 |
| Faith, church or religion (e.g., doubt, conflicts with your minister) , median (IQR) | 0 (0-0) | 0 (0-0) | 0 (0-0) | 0 (0-0) |
| Not stressful | 97.3 | 97.3 | 97.5 | 94.6 |
| Slightly stressful | 2.4 | 2.5 | 2.1 | 4.9 |
| Very stressful | 0.1 | 0.2 | 0.2 | 0.1 |
| Missing | 0.2 | 0.1 | 0.2 | 0.5 |

IQR: interquartile range Q1-Q3; ^a^ Chronic stress sum score and highlighted domains (work, partner, finances) displayed; ^b^ % presented, unless otherwise indicated; ^c^ Sum score of questionnaires T2, T3 and T4 for each Long-term Difficulties Inventory domain, % presented per category ‘not stressful’ indicates sum score 0; ‘slightly stressful’ sum score 1-3; ‘very stressful’ sum score 4-6.

**Supplementary Table 3. Baseline characteristics of the baseline population (n=120,177) and a comparison of the study population (n=53,216) and the participants excluded (n=66,961).**

| **Characteristics** | **Baseline population (n=120,177)^a^** | **Study population (n=53,216)^a,b^** | **Excluded population (n=66,961)^a^** | **Differences between study population and participants excluded^c^** |
| --- | --- | --- | --- | --- |
|  |  |  |  |  |
| **Demographic** |  |  |  |  |
| Age (years), mean (SD) | 43.0 (12.4) | 45.2 (12.2) | 41.2 (12.2) | 4.0 (3.9-4.2) |
| Sex (female) | 60.3 | 61.6 | 59.3 | 2.3 |
|  |  |  |  |  |
| **Socioeconomic** |  |  |  |  |
| Education (years), mean (SD) | 12.3 (2.4) | 12.3 (2.4) | 12.3 (2.4) | 0.0 (0.0-0.1) |
| Low^d^ | 26.6 | 27.1 | 26.2 | 0.9 |
| Middle^d^ | 41.1 | 39.8 | 42.1 | -2.3 |
| High^d^ | 32.3 | 33.1 | 31.7 | 1.4 |
| Occupational prestige (SIOPS08), mean (SD) | 43.5 (13.5) | 43.9 (13.4) | 43.2 (13.5) | 0.7 (0.5-0.8) |
| Equivalized household income (euros), mean (SD) | 1531.4 (579.2) | 1573.8 (571.3) | 1497.2 (583.3) | 76.7 (69.5-83.8) |
|  |  |  |  |  |
| **Metabolic syndrome indicators, meeting condition^e^** |  |  |  |  |
| Waist circumference^f^ | 26.0 | 26.3 | 25.8 | 0.5 |
| Triglyceride level^g^ | 8.7 | 8.6 | 8.8 | -0.2 |
| HDL cholesterol^h^ | 9.7 | 9.1 | 10.1 | -1 |
| Blood pressure^i^ | 29.3 | 31.6 | 27.4 | 4.2 |
| Glucose level^j^ | 5.5 | 5.7 | 5.3 | 0.4 |
|  |  |  |  |  |
| **Long-term difficulties** |  |  |  |  |
| Score on long-term difficulty inventory (LDI), mean (SD) | 2.5 (2.4) | 2.3 (2.3) | 2.7 (2.5) | -0.4 (-0.5-0.4) |
| Home and living (e.g., accommodation too small, could not find a home, noise), median (IQR) | 0 (0-0) | 0 (0-0) | 0 (0-0) | 0 (0-0) |
| Not stressful | 86.4 | 87.8 | 85.3 | 2.5 |
| Slightly stressful | 11.3 | 10.4 | 12.1 | -1.7 |
| Very stressful | 2.2 | 1.8 | 2.6 | -0.8 |
| At or with work (e.g., too demanding, conflicts with boss, (imminent) dismissal), median (IQR) | 0 (0-1) | 0 (0-1) | 0 (0-1) | 0 (0-0) |
| Not stressful | 62.0 | 63.2 | 61.0 | 2.2 |
| Slightly stressful | 30.5 | 29.9 | 31.0 | -1.1 |
| Very stressful | 7.5 | 6.9 | 8.0 | -1.1 |
| Relationship with friends or acquaintances (e.g., quarrels, lack of support), median (IQR) | 0 (0-0) | 0 (0-0) | 0 (0-0) | 0 (0-0) |
| Not stressful | 85.6 | 87.8 | 83.8 | 4 |
| Slightly stressful | 13.2 | 11.2 | 14.7 | -3.5 |
| Very stressful | 1.3 | 1.0 | 1.5 | -0.5 |
| Relationship with your partner (e.g., jealousy, conflicts, doubt about the relationship, quarrels), median (IQR) | 0 (0-0) | 0 (0-0) | 0 (0-0) | 0 (0-0) |
| Not stressful | 77.7 | 80.2 | 75.6 | 4.6 |
| Slightly stressful | 17.9 | 16.2 | 19.2 | -3 |
| Very stressful | 4.5 | 3.7 | 5.1 | -1.4 |
| Relationship with your children (e.g., frequent conflicts, lack of respect for you), median (IQR) | 0 (0-0) | 0 (0-0) | 0 (0-0) | 0 (0-0) |
| Not stressful | 83.7 | 83.8 | 83.6 | 0.2 |
| Slightly stressful | 14.7 | 14.7 | 14.7 | 0 |
| Very stressful | 1.6 | 1.5 | 1.7 | -0.2 |
| Relationship with your parents (e.g., frequent conflicts, lack of acceptance), median (IQR) | 0 (0-0) | 0 (0-0) | 0 (0-0) | 0 (0-0) |
| Not stressful | 86.2 | 88.1 | 84.7 | 3.4 |
| Slightly stressful | 11.9 | 10.4 | 13.0 | -2.6 |
| Very stressful | 1.9 | 1.5 | 2.2 | -0.7 |
| Relationship with other relatives (e.g., conflicts, lack of acceptance), median (IQR) | 0 (0-0) | 0 (0-0) | 0 (0-0) | 0 (0-0) |
| Not stressful | 83.7 | 84.2 | 83.2 | 1 |
| Slightly stressful | 14.5 | 14.2 | 14.7 | -0.5 |
| Very stressful | 1.9 | 1.6 | 2.1 | -0.5 |
| Free time (e.g., too little or too much free time), median (IQR) | 0 (0-1) | 0 (0-1) | 0 (0-1) | 0 (0-0) |
| Not stressful | 63.7 | 66.5 | 61.5 | 5 |
| Slightly stressful | 32.8 | 30.7 | 34.5 | -3.8 |
| Very stressful | 3.5 | 2.8 | 4.0 | -1.2 |
| Finances (e.g., major debts, insufficient income), median (IQR) | 0 (0-0) | 0 (0-0) | 0 (0-0) | 0 (0-0) |
| Not stressful | 82.4 | 85.9 | 79.7 | 6.2 |
| Slightly stressful | 15.0 | 12.4 | 17.2 | -4.8 |
| Very stressful | 2.6 | 1.7 | 3.2 | -1.5 |
| Your health (e.g., regularly ill, longer-term disorders), median (IQR) | 0 (0-0) | 0 (0-0) | 0 (0-0) | 0 (0-0) |
| Not stressful | 78.8 | 80.0 | 77.8 | 2.2 |
| Slightly stressful | 17.7 | 16.9 | 18.2 | -1.3 |
| Very stressful | 3.6 | 3.1 | 3.9 | -0.8 |
| School/study (too difficult, cannot be combined with other tasks), median (IQR) | 0 (0-0) | 0 (0-0) | 0 (0-0) | 0 (0-0) |
| Not stressful | 89.8 | 91.7 | 88.3 | 3.4 |
| Slightly stressful | 8.6 | 7.2 | 9.8 | -2.6 |
| Very stressful | 1.5 | 1.1 | 1.9 | -0.8 |
| Faith, church or religion (e.g., doubt, conflicts with your minister), median (IQR) | 0 (0-0) | 0 (0-0) | 0 (0-0) | 0 (0-0) |
| Not stressful | 96.7 | 96.4 | 96.8 | -0.4 |
| Slightly stressful | 3.1 | 3.4 | 2.9 | 0.5 |
| Very stressful | 0.3 | 0.2 | 0.3 | -0.1 |
|  |  |  |  |  |
| **Covariates related to Long-term Difficulties Inventory domains** |  |  |  |  |
| Work (yes) | 82.4 | 81.0 | 83.5 | -2.5 |
| Partner (yes) | 85.9 | 87.1 | 84.9 | 2.2 |
| Children (yes) | 73.9 | 75.6 | 72.2 | 3.4 |
| Parents died (yes) | 18.5 | 22.5 | 15.3 | 7.2 |
| School/study (yes) | 7.1 | 5.6 | 8.4 | 2.8 |
| Member of a church or other religious community (yes) | 19.7 | 22.0 | 17.8 | 4.2 |

SD: standard deviation; SIOPS08: Standard International Occupational Prestige Scale 2008; IQR: interquartile range Q1-Q3; HDL: high-density lipoprotein; ^a^ % presented, unless otherwise indicated; ^b^ Percentages might differ from Table 1 because missing values are not included and presented in the current table; ^c^ % or mean difference (99% Confidence Interval) presented; ^d^ Categories according to Dutch Standard Education Format [33]; ^e^ According to definition of metabolic syndrome by NCEP-ATPIII; ^f^ ≥ 102 cm in male, or ≥ 88 cm in female; ^g^ ≥ 1.70 mmol/l, or use of medication for elevated triglycerides; ^h^ < 1.0 mmol/L in male, < 1.3 mmol/L in female, or use of lipid-lowering medication; ^i^ Systolic blood pressure ≥ 130 mmHg, diastolic blood pressure ≥ 85 mmHg, or use of blood pressure-lowering medication; ^j^ Fasting blood glucose level ≥ 5.6 mmol/l, diagnosis of type 2 diabetes, or use of blood glucose-lowering medication.

**Supplementary Table 4. Baseline characteristics of the Lifelines Cohort Study (n=152,728) and a comparison of the study population (n=53,216) and the participants excluded (n=99,512).**

| **Characteristics** | **Lifelines Cohort Study population (n=152,728)^a^** | **Study population (n=53,216)^a,b^** | **Excluded population (n=99,512)^a^** | **Differences between study population and participants excluded^c^** |
| --- | --- | --- | --- | --- |
|  |  |  |  |  |
| **Demographic** |  |  |  |  |
| Age (years), mean (SD) | 44.6 (13.1) | 45.2 (12.2) | 44.3 (13.6) | -0.9 (-1.1- -0.8) |
| Sex (female) | 58.5 | 61.6 | 56.9 | 4.7 |
|  |  |  |  |  |
| **Socioeconomic** |  |  |  |  |
| Education (years), mean (SD) | 12.1 (2.5) | 12.3 (2.4) | 12.0 (2.5) | -0.4 (-0.4- -0.4) |
| Low^d^ | 30.6 | 27.1 | 32.4 | -5.3 |
| Middle^d^ | 39.5 | 39.8 | 39.4 | 0.4 |
| High^d^ | 29.9 | 33.1 | 28.2 | 4.9 |
| Occupational prestige (SIOPS08), mean (SD) | 43.0 (13.5) | 43.9 (13.4) | 42.6 (13.6) | -1.3 (-1.5- -1.2) |
| Equivalized household income (euros), mean (SD) | 1529.5 (578.7) | 1573.8 (571.3) | 1504.3 (581.4) | -69.5 (-76.2- -62.9) |
|  |  |  |  |  |
| **Metabolic syndrome indicators, meeting condition^e^** |  |  |  |  |
| Waist circumference^f^ | 35.1 | 26.3 | 39.8 | -13.5 |
| Triglyceride level^g^ | 20.2 | 8.6 | 26.7 | -18.1 |
| HDL cholesterol^h^ | 19.8 | 9.1 | 25.8 | -16.7 |
| Blood pressure^i^ | 37.1 | 31.6 | 40.0 | -8.4 |
| Glucose level^j^ | 13.1 | 5.7 | 17.2 | -11.5 |
|  |  |  |  |  |
| **Long-term difficulties** |  |  |  |  |
| Score on long-term difficulty inventory (LDI), mean (SD) | 2.5 (2.4) | 2.3 (2.3) | 2.6 (2.5) | 0.3 (0.3-0.3) |
| Home and living (e.g., accommodation too small, could not find a home, noise), median (IQR) | 0 (0-0) | 0 (0-0) | 0 (0-0) | 0 (0-0) |
| Not stressful | 87.1 | 87.8 | 86.7 | 1.1 |
| Slightly stressful | 10.7 | 10.4 | 11.0 | -0.6 |
| Very stressful | 2.2 | 1.8 | 2.4 | -0.6 |
| At or with work (e.g., too demanding, conflicts with boss, (imminent) dismissal), median (IQR) | 0 (0-1) | 0 (0-1) | 0 (0-1) | 0 (0-0) |
| Not stressful | 63.5 | 63.2 | 63.7 | -0.5 |
| Slightly stressful | 29.2 | 29.9 | 28.7 | 1.2 |
| Very stressful | 7.3 | 6.9 | 7.6 | 0.7 |
| Relationship with friends or acquaintances (e.g., quarrels, lack of support), median (IQR) | 0 (0-0) | 0 (0-0) | 0 (0-0) | 0 (0-0) |
| Not stressful | 85.9 | 87.8 | 84.8 | 3.0 |
| Slightly stressful | 12.9 | 11.2 | 13.8 | -2.6 |
| Very stressful | 1.3 | 1.0 | 1.4 | -0.4 |
| Relationship with your partner (e.g., jealousy, conflicts, doubt about the relationship, quarrels), median (IQR) | 0 (0-0) | 0 (0-0) | 0 (0-0) | 0 (0-0) |
| Not stressful | 78.7 | 80.2 | 77.9 | 2.3 |
| Slightly stressful | 17.1 | 16.2 | 17.6 | -1.4 |
| Very stressful | 4.2 | 3.7 | 4.5 | -0.8 |
| Relationship with your children (e.g., frequent conflicts, lack of respect for you), median (IQR) | 0 (0-0) | 0 (0-0) | 0 (0-0) | 0 (0-0) |
| Not stressful | 83.6 | 83.8 | 83.5 | 0.3 |
| Slightly stressful | 14.7 | 14.7 | 14.7 | 0 |
| Very stressful | 1.7 | 1.5 | 1.8 | -0.3 |
| Relationship with your parents (e.g., frequent conflicts, lack of acceptance), median (IQR) | 0 (0-0) | 0 (0-0) | 0 (0-0) | 0 (0-0) |
| Not stressful | 86.8 | 88.1 | 86.1 | 2.0 |
| Slightly stressful | 11.3 | 10.4 | 11.8 | -1.4 |
| Very stressful | 1.9 | 1.5 | 2.1 | -0.6 |
| Relationship with other relatives (e.g., conflicts, lack of acceptance), median (IQR) | 0 (0-0) | 0 (0-0) | 0 (0-0) | 0 (0-0) |
| Not stressful | 83.7 | 84.2 | 83.4 | 0.8 |
| Slightly stressful | 14.4 | 14.2 | 14.5 | -0.3 |
| Very stressful | 1.9 | 1.6 | 2.1 | -0.5 |
| Free time (e.g., too little or too much free time), median (IQR) | 0 (0-1) | 0 (0-1) | 0 (0-1) | 0 (0-0) |
| Not stressful | 64.9 | 66.5 | 64.0 | 2.5 |
| Slightly stressful | 31.7 | 30.7 | 32.3 | -1.6 |
| Very stressful | 3.4 | 2.8 | 3.7 | -0.9 |
| Finances (e.g., major debts, insufficient income), median (IQR) | 0 (0-0) | 0 (0-0) | 0 (0-0) | 0 (0-0) |
| Not stressful | 82.3 | 85.9 | 80.2 | 5.7 |
| Slightly stressful | 15.0 | 12.4 | 16.5 | -4.1 |
| Very stressful | 2.7 | 1.7 | 3.2 | -1.5 |
| Your health (e.g., regularly ill, longer-term disorders), median (IQR) | 0 (0-0) | 0 (0-0) | 0 (0-0) | 0 (0-0) |
| Not stressful | 77.3 | 80.0 | 75.7 | 4.3 |
| Slightly stressful | 18.8 | 16.9 | 19.9 | -3.0 |
| Very stressful | 3.9 | 3.1 | 4.4 | -1.3 |
| School/study (too difficult, cannot be combined with other tasks), median (IQR) | 0 (0-0) | 0 (0-0) | 0 (0-0) | 0 (0-0) |
| Not stressful | 90.7 | 91.7 | 90.1 | 1.6 |
| Slightly stressful | 7.9 | 7.2 | 8.3 | -1.1 |
| Very stressful | 1.4 | 1.1 | 1.6 | -0.5 |
| Faith, church or religion (e.g., doubt, conflicts with your minister), median (IQR) | 0 (0-0) | 0 (0-0) | 0 (0-0) | 0 (0-0) |
| Not stressful | 96.6 | 96.4 | 96.7 | -0.3 |
| Slightly stressful | 3.1 | 3.4 | 3.0 | 0.4 |
| Very stressful | 0.3 | 0.2 | 0.3 | -0.1 |
|  |  |  |  |  |
| **Covariates related to Long-term Difficulties Inventory domains** |  |  |  |  |
| Work (yes) | 79.7 | 81.0 | 79.0 | 2.0 |
| Partner (yes) | 85.5 | 87.1 | 84.7 | 2.4 |
| Children (yes) | 75.5 | 75.6 | 75.5 | 0.1 |
| Parents died (yes) | 21.0 | 22.5 | 20.2 | 2.3 |
| School/study (yes) | 6.3 | 5.6 | 6.7 | -1.1 |
| Member of a church or other religious community (yes) | 18.7 | 22.0 | 16.9 | 5.1 |

SD: standard deviation; SIOPS08: Standard International Occupational Prestige Scale 2008; IQR: interquartile range Q1-Q3; HDL: high-density lipoprotein; ^a^ % presented, unless otherwise indicated; ^b^ Percentages might differ from Table 1 because missing values are not included and presented in the current table; ^c^ % or mean difference (99% Confidence Interval) presented; ^d^ Categories according to Dutch Standard Education Format [33]; ^e^ According to definition of metabolic syndrome by NCEP-ATPIII; ^f^ ≥ 102 cm in male, or ≥ 88 cm in female; ^g^ ≥ 1.70 mmol/l, or use of medication for elevated triglycerides; ^h^ < 1.0 mmol/L in male, < 1.3 mmol/L in female, or use of lipid-lowering medication; ^i^ Systolic blood pressure ≥ 130 mmHg, diastolic blood pressure ≥ 85 mmHg, or use of blood pressure-lowering medication; ^j^ Fasting blood glucose level ≥ 5.6 mmol/l, diagnosis of type 2 diabetes, or use of blood glucose-lowering medication.

**Supplementary Table 5. Multivariable logistic and linear regression analysis of direct associations between socioeconomic position, chronic stress^a^, and metabolic syndrome development in the study population (n=53,216).**

|  | **Education** | **Occupational prestige** | **Income** |
| --- | --- | --- | --- |
|  | **OR (99% CI)** | **OR (99% CI)** | **OR (99% CI)** |
| **Path 1. SEP and MetS development** | 0.92 (0.90, 0.94)* | 0.95 (0.91, 0.99)* | 0.99 (0.98, 1.00) |
| **Path 2. SEP and chronic stress^a^** |  |  |  |
| Sum score (beta) | 0.23 (0.20, 0.26)* | 0.16 (0.11, 0.22)* | -0.10 (-0.11, -0.08)* |
| Related to home and living |  |  |  |
| Slightly stressful | 1.11 (1.09, 1.13)* | 1.01 (0.99, 1.04) | 0.98 (0.97, 0.99)* |
| Very stressful | 1.15 (1.09, 1.21)* | 0.95 (0.88, 1.04) | 0.95 (0.93, 0.97)* |
| At or with work |  |  |  |
| Slightly stressful | 1.14 (1.12, 1.15)* | 1.09 (1.07, 1.12)* | 1.01 (1.00, 1.01)* |
| Very stressful | 1.21 (1.18, 1.24)* | 1.17 (1.12, 1.21)* | 1.02 (1.01, 1.03)* |
| Related to relationship with friends or acquaintances |  |  |  |
| Slightly stressful | 1.00 (0.99, 1.02) | 1.00 (0.97, 1.03) | 0.98 (0.97, 0.98)* |
| Very stressful | 0.99 (0.92, 1.07) | 0.98 (0.87, 1.11) | 0.94 (0.91, 0.97)* |
| Related to relationship with partner |  |  |  |
| Slightly stressful | 1.06 (1.04, 1.08)* | 1.03 (1.01, 1.06)* | 0.98 (0.98, 0.99)* |
| Very stressful | 1.12 (1.08, 1.15)* | 1.04 (0.99, 1.10) | 0.97 (0.95, 0.98)* |
| Related to relationship with children |  |  |  |
| Slightly stressful | 1.05 (1.04, 1.07)* | 1.02 (0.99, 1.05) | 0.96 (0.95, 0.96)* |
| Very stressful | 1.05 (1.00, 1.10) | 1.09 (1.01, 1.18)* | 0.91 (0.89, 0.93)* |
| Related to relationship with parents |  |  |  |
| Slightly stressful | 1.01 (0.99, 1.03) | 1.00 (0.97, 1.03) | 0.99 (0.98, 0.99)* |
| Very stressful | 1.02 (0.97, 1.08) | 0.97 (0.89, 1.05) | 0.97 (0.95, 0.99)* |
| Related to relationship with relatives |  |  |  |
| Slightly stressful | 1.01 (1.00, 1.03) | 0.97 (0.95, 1.00)* | 0.99 (0.99, 1.00)* |
| Very stressful | 1.00 (0.95, 1.06) | 0.98 (0.89, 1.07) | 0.99 (0.97, 1.01) |
| Related to free time |  |  |  |
| Slightly stressful | 1.07 (1.05, 1.08)* | 1.05 (1.03, 1.07)* | 1.00 (0.99, 1.00) |
| Very stressful | 1.11 (1.07, 1.15)* | 1.13 (1.07, 1.20)* | 0.98 (0.96, 0.99)* |
| Related to finances |  |  |  |
| Slightly stressful | 1.05 (1.03, 1.07)* | 1.00 (0.97, 1.03) | 0.93 (0.92, 0.94)* |
| Very stressful | 1.05 (1.00, 1.10) | 1.00 (0.93, 1.09) | 0.88 (0.86, 0.90)* |
| Related to own health |  |  |  |
| Slightly stressful | 1.02 (1.01, 1.04)* | 1.02 (1.00, 1.04) | 0.99 (0.99, 1.00)* |
| Very stressful | 1.01 (0.97, 1.04) | 1.04 (0.98, 1.11) | 0.98 (0.96, 0.99)* |
| Related to school/study |  |  |  |
| Slightly stressful | 1.06 (1.04, 1.09)* | 1.10 (1.06, 1.14)* | 0.99 (0.98, 1.00)* |
| Very stressful | 1.05 (0.98, 1.12) | 1.11 (1.00, 1.24)* | 0.97 (0.94, 1.00)* |
| Related to faith, church or religion |  |  |  |
| Slightly stressful | 1.02 (0.98, 1.05) | 1.02 (0.97, 1.08) | 0.97 (0.95, 0.98)* |
| Very stressful | 1.17 (0.99, 1.39) | 0.85 (0.63, 1.15) | 0.95 (0.88, 1.02) |
| **Path 3. Chronic stress^a^ and MetS development** |  |  |  |
| Sum score | 1.02 (1.01, 1.03)* | 1.02 (1.01, 1.03)* | 1.02 (1.01, 1.03)* |
| Related to home and living |  |  |  |
| Slightly stressful | 0.88 (0.77, 1.00) | 0.88 (0.77, 1.00) | 0.88 (0.77, 1.00) |
| Very stressful | 0.84 (0.57, 1.25) | 0.84 (0.57, 1.25) | 0.84 (0.57, 1.25) |
| At or with work |  |  |  |
| Slightly stressful | 0.99 (0.89, 1.10) | 0.99 (0.89, 1.10) | 0.99 (0.89, 1.10) |
| Very stressful | 1.07 (0.89, 1.28) | 1.07 (0.89, 1.28) | 1.07 (0.89, 1.28) |
| Related to relationship with friends or acquaintances |  |  |  |
| Slightly stressful | 1.07 (0.95, 1.21) | 1.07 (0.95, 1.21) | 1.07 (0.95, 1.21) |
| Very stressful | 1.35 (0.83, 2.19) | 1.35 (0.83, 2.19) | 1.35 (0.83, 2.19) |
| Related to relationship with partner |  |  |  |
| Slightly stressful | 0.82 (0.73, 0.92)* | 0.82 (0.73, 0.92)* | 0.82 (0.73, 0.92)* |
| Very stressful | 0.80 (0.62, 1.05) | 0.80 (0.62, 1.05) | 0.80 (0.62, 1.05) |
| Related to relationship with children |  |  |  |
| Slightly stressful | 1.03 (0.92, 1.15) | 1.03 (0.92, 1.15) | 1.03 (0.92, 1.15) |
| Very stressful | 0.97 (0.70, 1.34) | 0.97 (0.70, 1.34) | 0.97 (0.70, 1.34) |
| Related to relationship with parents |  |  |  |
| Slightly stressful | 0.97 (0.84, 1.11) | 0.97 (0.84, 1.11) | 0.97 (0.84, 1.11) |
| Very stressful | 1.24 (0.87, 1.76) | 1.24 (0.87, 1.76) | 1.24 (0.87, 1.76) |
| Related to relationship with relatives |  |  |  |
| Slightly stressful | 1.04 (0.93, 1.16) | 1.04 (0.93, 1.16) | 1.04 (0.93, 1.16) |
| Very stressful | 1.10 (0.76, 1.60) | 1.10 (0.76, 1.60) | 1.10 (0.76, 1.60) |
| Related to free time |  |  |  |
| Slightly stressful | 1.00 (0.90, 1.10) | 1.00 (0.90, 1.10) | 1.00 (0.90, 1.10) |
| Very stressful | 1.02 (0.78, 1.33) | 1.02 (0.78, 1.33) | 1.02 (0.78, 1.33) |
| Related to finances |  |  |  |
| Slightly stressful | 1.33 (1.18, 1.50)* | 1.33 (1.18, 1.50)* | 1.33 (1.18, 1.50)* |
| Very stressful | 1.58 (1.16, 2.15)* | 1.58 (1.16, 2.15)* | 1.58 (1.16, 2.15)* |
| Related to own health |  |  |  |
| Slightly stressful | 1.32 (1.20, 1.46)* | 1.32 (1.20, 1.46)* | 1.32 (1.20, 1.46)* |
| Very stressful | 1.82 (1.47, 2.25)* | 1.82 (1.47, 2.25)* | 1.82 (1.47, 2.25)* |
| Related to school/study |  |  |  |
| Slightly stressful | 0.99 (0.83, 1.17) | 0.99 (0.83, 1.17) | 0.99 (0.83, 1.17) |
| Very stressful | 0.95 (0.52, 1.74) | 0.95 (0.52, 1.74) | 0.95 (0.52, 1.74) |
| Related to faith, church or religion |  |  |  |
| Slightly stressful | 1.02 (0.83, 1.26) | 1.02 (0.83, 1.26) | 1.02 (0.83, 1.26) |
| Very stressful | 1.33 (0.47, 3.80) | 1.33 (0.47, 3.80) | 1.33 (0.47, 3.80) |
| **Path 4. SEP and MetS development^b^** |  |  |  |
| Sum score | 0.92 (0.90, 0.94)* | 0.95 (0.91, 0.99)* | 1.00 (0.99, 1.01) |
| Related to home and living | 0.92 (0.90, 0.94)* | 0.95 (0.91, 0.99)* | 0.99 (0.98, 1.00) |
| At or with work | 0.92 (0.90, 0.94)* | 0.95 (0.91, 0.99)* | 0.99 (0.98, 1.00) |
| Related to relationship with friends or acquaintances | 0.92 (0.90, 0.94)* | 0.95 (0.91, 0.99)* | 0.99 (0.98, 1.00) |
| Related to relationship with partner | 0.92 (0.90, 0.94)* | 0.95 (0.91, 0.99)* | 0.99 (0.98, 1.00) |
| Related to relationship with children | 0.92 (0.90, 0.94)* | 0.95 (0.91, 0.99)* | 0.99 (0.98, 1.00) |
| Related to relationship with parents | 0.92 (0.90, 0.94)* | 0.95 (0.91, 0.99)* | 0.99 (0.98, 1.00) |
| Related to relationship with relatives | 0.92 (0.90, 0.94)* | 0.95 (0.91, 0.99)* | 0.99 (0.98, 1.00) |
| Related to free time | 0.92 (0.90, 0.94)* | 0.95 (0.91, 0.99)* | 0.99 (0.98, 1.00) |
| Related to finances | 0.92 (0.90, 0.94)* | 0.95 (0.91, 0.99)* | 1.00 (0.99, 1.01) |
| Related to own health | 0.92 (0.90, 0.94)* | 0.95 (0.91, 0.99)* | 0.99 (0.98, 1.00) |
| Related to school/study | 0.92 (0.90, 0.94)* | 0.95 (0.91, 0.99)* | 0.99 (0.98, 1.00) |
| Related to faith, church or religion | 0.92 (0.90, 0.94)* | 0.95 (0.91, 0.99)* | 0.99 (0.98, 1.00) |
| OR: odds ratio; CI: confidence interval; SEP: socioeconomic position; MetS: metabolic syndrome; LDI: Long-term Difficulties Inventory; ^a^ Long-term difficulties during total follow-up time measured with the LDI, LDI categories consist of the sum score of the LDI from questionnaires T2, T3 and T4, ‘not stressful’ indicates sum score 0, ‘slightly stressful’ sum score 1-3, ‘very stressful’ sum score 4-6; ^b^ Direct associations between SEP measures and MetS development controlled for specific LDI domain; analyses controlled for years of education, equivalized household income, occupational prestige, age and sex at T1, and time between T1 and T4; reference category for the LDI domains was ‘not stressful’; LDI domains were controlled for work status, partner status, children status, parent status, school/study status and religion status where applicable; *P<0.01. | | | |

**Supplementary Table 6. Multivariable mediation analysis of chronic stress^a^ in associations between socioeconomic position and metabolic syndrome development, using the Karlson-Holm-Breen method in the study population (n=53,216).**

|  | **Education** | **Occupational prestige** | **Income** |
| --- | --- | --- | --- |
|  | **OR (99% CI)** | **OR (99% CI)** | **OR (99% CI)** |
| **Total association** | 0.92 (0.90, 0.94)* | 0.95 (0.91, 0.99)* | 0.99 (0.98, 1.00) |
| **Direct association** | 0.92 (0.90, 0.94)* | 0.95 (0.91, 0.99)* | 1.00 (0.99, 1.00) |
| **Indirect association** | 1.00 (1.00, 1.01)* | 1.00 (1.00, 1.00)* | 1.00 (1.00, 1.00) |
|  |  |  |  |
|  | **Percentage** | **Percentage** | **Percentage** |
| **Mediating effect** |  |  |  |
| Sum score | -5.6 | -6.2 | 25.1 |
|  |  |  |  |
| **Mediating effects per domain of life** |  |  |  |
| Related to home and living | 2.3 | 0.7 | -9.0 |
| At or with work | -0.5 | -1.0 | -1.1 |
| Related to relationship with friends or acquaintances | -0.1 | 0.0 | 4.6 |
| Related to relationship with partner | 2.7 | 2.9 | -12.2 |
| Related to relationship with children | -0.2 | 0.0 | 1.4 |
| Related to relationship with parents | 0.0 | 0.2 | 0.5 |
| Related to relationship with relatives | -0.1 | 0.4 | 1.0 |
| Related to free time | 0.0 | -0.1 | 0.1 |
| Related to finances | -2.3 | 0.4 | 51.5 |
| Related to own health | -1.7 | -3.6 | 12.6 |
| Related to school/study | 0.1 | 0.3 | -0.8 |
| Related to faith, church or religion | -0.1 | -0.1 | 1.9 |
| OR: odds ratio; CI: confidence interval; SEP: socioeconomic position; MetS: metabolic syndrome; LDI: Long-term Difficulties Inventory; ^a^ Long-term difficulties during total follow-up time measured with the LDI; analyses controlled for years of education, equivalized household income, occupational prestige, age and sex at T1, and time between T1 and T4; LDI domains were controlled for work status, partner status, children status, parent status, school/study status and religion status where applicable; *P<0.01. | | | |

**Supplementary Table 7. Interaction coefficients of sex*chronic stress in the multivariable logistic regression analysis between chronic stress^a^ and metabolic syndrome development (n=53,216).**

| **Chronic stress^a^ domain** | **OR (99% CI)** |
| --- | --- |
| Female*Sum score | 0.99 (0.98, 1.01) |
| Related to home and living |  |
| Female*Not stressful | 1.00 |
| Female*Slightly stressful | 0.82 (0.64, 1.04) |
| Female*Very stressful | 0.68 (0.31, 1.49) |
| At or with work |  |
| Female*Not stressful | 1.00 |
| Female*Slightly stressful | 0.89 (0.74, 1.07) |
| Female*Very stressful | 0.93 (0.67, 1.30) |
| Related to relationship with friends |  |
| Female*Not stressful | 1.00 |
| Female*Slightly stressful | 0.96 (0.77, 1.20) |
| Female*Very stressful | 1.48 (0.47, 4.71) |
| Related to relationship with partner |  |
| Female*Not stressful | 1.00 |
| Female*Slightly stressful | 0.89 (0.71, 1.10) |
| Female*Very stressful | 0.90 (0.53, 1.52) |
| Related to relationship with children |  |
| Female*Not stressful | 1.00 |
| Female*Slightly stressful | 1.05 (0.85, 1.29) |
| Female*Very stressful | 0.97 (0.49, 1.93) |
| Related to relationship with parents |  |
| Female*Not stressful | 1.00 |
| Female*Slightly stressful | 0.89 (0.68, 1.15) |
| Female*Very stressful | 0.83 (0.39, 1.74) |
| Related to relationship with other relatives |  |
| Female*Not stressful | 1.00 |
| Female*Slightly stressful | 1.08 (0.87, 1.33) |
| Female*Very stressful | 0.83 (0.38, 1.82) |
| Related to free time |  |
| Female*Not stressful | 1.00 |
| Female*Slightly stressful | 0.86 (0.72, 1.03) |
| Female*Very stressful | 0.68 (0.41, 1.12) |
| Related to finances |  |
| Female*Not stressful | 1.00 |
| Female*Slightly stressful | 0.89 (0.72, 1.11) |
| Female*Very stressful | 1.26 (0.68, 2.34) |
| Related to your health |  |
| Female*Not stressful | 1.00 |
| Female*Slightly stressful | 1.03 (0.85, 1.24) |
| Female*Very stressful | 1.06 (0.68, 1.65) |
| Related to school/study |  |
| Female*Not stressful | 1.00 |
| Female*Slightly stressful | 0.90 (0.65, 1.26) |
| Female*Very stressful | 0.54 (0.17, 1.73) |
| Related to faith, church or religion |  |
| Female*Not stressful | 1.00 |
| Female*Slightly stressful | 0.94 (0.63, 1.41) |
| Female*Very stressful | 3.47 (0.36, 33.78) |
| OR: odds ratio; CI: confidence interval; LDI: Long-term Difficulties Inventory; ^a^ Long-term difficulties during total follow-up time measured with the LDI, LDI categories consist of the sum score of the LDI from questionnaires T2, T3 and T4, ‘not stressful’ indicates sum score 0, ‘slightly stressful’ sum score 1-3, ‘very stressful’ sum score 4-6; analyses controlled for years of education, equivalized household income, occupational prestige, age and sex at T1; reference category was ‘male’; LDI domains were controlled for work status, partner status, children status, parent status, school/study status, and religion status where applicable; *P<0.01. | |

**Supplementary Table 8. Multivariable logistic and linear regression analysis of direct associations between socioeconomic position, chronic stress^a^, and metabolic syndrome development among participants who did not use medication at T4 (n=31,358).**

|  | **Education** | **Occupational prestige** | **Income** |
| --- | --- | --- | --- |
|  | **OR (99% CI)** | **OR (99% CI)** | **OR (99% CI)** |
| **Path 1. SEP and MetS development** | 0.92 (0.89, 0.95)* | 0.98 (0.92, 1.04) | 0.99 (0.98, 1.00) |
| **Path 2. SEP and chronic stress^a^** |  |  |  |
| Sum score (beta) | 0.24 (0.20, 0.28)* | 0.19 (0.12, 0.26)* | -0.10 (-0.11, -0.08) |
| Related to home and living |  |  |  |
| Slightly stressful | 1.11 (1.09, 1.14)* | 1.02 (0.98, 1.06) | 0.98 (0.97, 0.99)* |
| Very stressful | 1.20 (1.11, 1.29)* | 0.94 (0.83, 1.05) | 0.96 (0.93, 0.98)* |
| At or with work |  |  |  |
| Slightly stressful | 1.14 (1.12, 1.16)* | 1.10 (1.06, 1.13)* | 1.01 (1.00, 1.02)* |
| Very stressful | 1.23 (1.19, 1.27)* | 1.17 (1.11, 1.24)* | 1.02 (1.01, 1.03)* |
| Related to relationship with friends or acquaintances |  |  |  |
| Slightly stressful | 1.00 (0.98, 1.03) | 1.02 (0.99, 1.06) | 0.98 (0.97, 0.98)* |
| Very stressful | 0.99 (0.89, 1.10) | 0.99 (0.83, 1.18) | 0.94 (0.91, 0.98)* |
| Related to relationship with partner |  |  |  |
| Slightly stressful | 1.05 (1.03, 1.07)* | 1.05 (1.02, 1.08)* | 0.98 (0.98, 0.99)* |
| Very stressful | 1.13 (1.08, 1.18)* | 1.05 (0.98, 1.13) | 0.97 (0.95, 0.98)* |
| Related to relationship with children |  |  |  |
| Slightly stressful | 1.05 (1.03, 1.07)* | 1.02 (0.98, 1.05) | 0.96 (0.95, 0.96)* |
| Very stressful | 1.04 (0.97, 1.10) | 1.16 (1.04, 1.29)* | 0.91 (0.89, 0.94)* |
| Related to relationship with parents |  |  |  |
| Slightly stressful | 1.01 (0.99, 1.03) | 1.00 (0.97, 1.04) | 0.98 (0.98, 0.99)* |
| Very stressful | 1.01 (0.94, 1.09) | 0.98 (0.87, 1.11) | 0.96 (0.93, 0.98)* |
| Related to relationship with relatives |  |  |  |
| Slightly stressful | 1.01 (0.99, 1.03) | 0.99 (0.96, 1.02) | 0.99 (0.98, 1.00)* |
| Very stressful | 1.02 (0.94, 1.10) | 1.02 (0.90, 1.16) | 0.99 (0.96, 1.02) |
| Related to free time |  |  |  |
| Slightly stressful | 1.07 (1.05, 1.09)* | 1.06 (1.03, 1.09)* | 0.99 (0.99, 1.00) |
| Very stressful | 1.09 (1.04, 1.15)* | 1.14 (1.06, 1.24)* | 0.97 (0.96, 0.99)* |
| Related to finances |  |  |  |
| Slightly stressful | 1.05 (1.03, 1.08)* | 1.00 (0.96, 1.03) | 0.93 (0.92, 0.94)* |
| Very stressful | 1.06 (0.99, 1.13) | 0.97 (0.87-1.09) | 0.89 (0.87, 0.92)* |
| Related to own health |  |  |  |
| Slightly stressful | 1.04 (1.02, 1.06)* | 1.03 (1.00, 1.06) | 1.00 (0.99, 1.00) |
| Very stressful | 0.98 (0.91, 1.05) | 1.07 (0.96, 1.20) | 0.99 (0.97, 1.02) |
| Related to school/study |  |  |  |
| Slightly stressful | 1.07 (1.04, 1.10)* | 1.09 (1.04, 1.14)* | 0.98 (0.97, 0.99)* |
| Very stressful | 1.07 (0.98, 1.17) | 1.10 (0.96, 1.26) | 0.96 (0.93, 1.00)* |
| Related to faith, church or religion |  |  |  |
| Slightly stressful | 1.00 (0.96, 1.04) | 1.04 (0.97, 1.11) | 0.97 (0.95, 0.98)* |
| Very stressful | 1.12 (0.88, 1.43) | 0.90 (0.59, 1.38) | 0.97 (0.88, 1.07) |
| **Path 3. Chronic stress^a^ and MetS development** |  |  |  |
| Sum score | 1.01 (1.00, 1.02) | 1.01 (1.00, 1.02) | 1.01 (1.00, 1.02) |
| Related to home and living |  |  |  |
| Slightly stressful | 0.95 (0.79, 1.15) | 0.95 (0.79, 1.15) | 0.95 (0.79, 1.15) |
| Very stressful | 0.84 (0.43, 1.64) | 0.84 (0.43, 1.64) | 0.84 (0.43, 1.64) |
| At or with work |  |  |  |
| Slightly stressful | 0.93 (0.80, 1.09) | 0.93 (0.80, 1.09) | 0.93 (0.80, 1.09) |
| Very stressful | 0.86 (0.65, 1.14) | 0.86 (0.65, 1.14) | 0.86 (0.65, 1.14) |
| Related to relationship with friends or acquaintances |  |  |  |
| Slightly stressful | 1.10 (0.92, 1.32) | 1.10 (0.92, 1.32) | 1.10 (0.92, 1.32) |
| Very stressful | 1.13 (0.48, 2.67) | 1.13 (0.48, 2.67) | 1.13 (0.48, 2.67) |
| Related to relationship with partner |  |  |  |
| Slightly stressful | 0.83 (0.70, 0.98)* | 0.83 (0.70, 0.98)* | 0.83 (0.70, 0.98)* |
| Very stressful | 0.86 (0.58, 1.27) | 0.86 (0.58, 1.27) | 0.86 (0.58, 1.27) |
| Related to relationship with children |  |  |  |
| Slightly stressful | 1.03 (0.87, 1.22) | 1.03 (0.87, 1.22) | 1.03 (0.87, 1.22) |
| Very stressful | 1.00 (0.60, 1.65) | 1.00 (0.60, 1.65) | 1.00 (0.60, 1.65) |
| Related to relationship with parents |  |  |  |
| Slightly stressful | 0.98 (0.80, 1.20) | 0.98 (0.80, 1.20) | 0.98 (0.80, 1.20) |
| Very stressful | 0.93 (0.49, 1.76) | 0.93 (0.49, 1.76) | 0.93 (0.49, 1.76) |
| Related to relationship with relatives |  |  |  |
| Slightly stressful | 0.98 (0.82, 1.16) | 0.98 (0.82, 1.16) | 0.98 (0.82, 1.16) |
| Very stressful | 1.14 (0.61, 2.10) | 1.14 (0.61, 2.10) | 1.14 (0.61, 2.10) |
| Related to free time |  |  |  |
| Slightly stressful | 1.00 (0.87, 1.16) | 1.00 (0.87, 1.16) | 1.00 (0.87, 1.16) |
| Very stressful | 0.94 (0.62, 1.41) | 0.94 (0.62, 1.41) | 0.94 (0.62, 1.41) |
| Related to finances |  |  |  |
| Slightly stressful | 1.39 (1.17, 1.65)* | 1.39 (1.17, 1.65)* | 1.39 (1.17, 1.65)* |
| Very stressful | 1.19 (0.69, 2.05) | 1.19 (0.69, 2.05) | 1.19 (0.69, 2.05) |
| Related to own health |  |  |  |
| Slightly stressful | 1.26 (1.08, 1.47)* | 1.26 (1.08, 1.47)* | 1.26 (1.08, 1.47)* |
| Very stressful | 1.38 (0.82, 2.34) | 1.38 (0.82, 2.34) | 1.38 (0.82, 2.34) |
| Related to school/study |  |  |  |
| Slightly stressful | 0.97 (0.75, 1.25) | 0.97 (0.75, 1.25) | 0.97 (0.75, 1.25) |
| Very stressful | 1.30 (0.58, 2.91) | 1.30 (0.58, 2.91) | 1.30 (0.58, 2.91) |
| Related to faith, church or religion |  |  |  |
| Slightly stressful | 0.97 (0.70, 1.35) | 0.97 (0.70, 1.35) | 0.97 (0.70, 1.35) |
| Very stressful | 0.99 (0.15-6.55) | 0.99 (0.15-6.55) | 0.99 (0.15-6.55) |
| **Path 4. SEP and MetS development^b^** |  |  |  |
| Sum score | 0.91 (0.88, 0.95)* | 0.98 (0.92, 1.04) | 0.99 (0.98, 1.00) |
| Related to home and living | 0.92 (0.89, 0.95)* | 0.98 (0.92, 1.05) | 0.99 (0.98, 1.00) |
| At or with work | 0.92 (0.89, 0.95)* | 0.98 (0.92, 1.05) | 0.99 (0.98, 1.00) |
| Related to relationship with friends or acquaintances | 0.92 (0.89, 0.95)* | 0.98 (0.92, 1.05) | 0.99 (0.98, 1.00) |
| Related to relationship with partner | 0.92 (0.89, 0.95)* | 0.98 (0.92, 1.05) | 0.99 (0.98, 1.00) |
| Related to relationship with children | 0.92 (0.89, 0.95)* | 0.98 (0.92, 1.05) | 0.99 (0.98, 1.00) |
| Related to relationship with parents | 0.92 (0.89, 0.95)* | 0.98 (0.92, 1.05) | 0.99 (0.98, 1.00) |
| Related to relationship with relatives | 0.92 (0.89, 0.95)* | 0.98 (0.92, 1.05) | 0.99 (0.98, 1.00) |
| Related to free time | 0.92 (0.89, 0.95)* | 0.98 (0.92, 1.05) | 0.99 (0.98, 1.00) |
| Related to finances | 0.91 (0.88, 0.95)* | 0.98 (0.92, 1.05) | 0.99 (0.98, 1.01) |
| Related to own health | 0.91 (0.88, 0.95)* | 0.98 (0.92, 1.04) | 0.99 (0.98, 1.00) |
| Related to school/study | 0.92 (0.89, 0.95)* | 0.98 (0.92, 1.05) | 0.99 (0.98, 1.00) |
| Related to faith, church or religion | 0.92 (0.89, 0.95)* | 0.98 (0.92, 1.05) | 0.99 (0.98, 1.00) |
| OR: odds ratio; CI: confidence interval; SEP: socioeconomic position; MetS: metabolic syndrome; LDI: Long-term Difficulties Inventory; ^a^ Long-term difficulties during total follow-up time measured with the LDI, LDI categories consist of the sum score of the LDI from questionnaires T2, T3 and T4, ‘not stressful’ indicates sum score 0, ‘slightly stressful’ sum score 1-3, ‘very stressful’ sum score 4-6; ^b^ Direct associations between SEP measures and MetS development controlled for specific LDI domain; analyses controlled for years of education, equivalized household income, occupational prestige, age and sex at T1, and time between T1 and T4; reference category for the LDI domains was ‘not stressful’; LDI domains were controlled for work status, partner status, children status, parent status, school/study status and religion status where applicable; *P<0.01. | | | |

**Supplementary Table 9. Multivariable mediation analysis of chronic stress^a^ in associations between socioeconomic position and metabolic syndrome development, using the Karlson-Holm-Breen method among participants who did not use medication at T4 (n=31,358).**

|  | **Education** | **Occupational prestige** | **Income** |
| --- | --- | --- | --- |
|  | **OR (99% CI)** | **OR (99% CI)** | **OR (99% CI)** |
| **Total association** | 0.92 (0.89, 0.95)* | 0.98 (0.92, 1.05) | 0.99 (0.98, 1.00) |
| **Direct association** | 0.91 (0.88, 0.95)* | 0.98 (0.92, 1.04) | 0.99 (0.98, 1.00) |
| **Indirect association** | 1.00 (1.00, 1.01) | 1.00 (1.00, 1.00) | 1.00 (1.00, 1.00) |
|  |  |  |  |
|  | **Percentage** | **Percentage** | **Percentage** |
| **Mediating effect** |  |  |  |
| Sum score | -1.9 | -7.9 | 7.6 |
|  |  |  |  |
| **Mediating effects per domain of life** |  |  |  |
| Related to home and living | 1.3 | 0.2 | -2.5 |
| At or with work | 2.3 | 9.7 | 3.0 |
| Related to relationship with friends or acquaintances | 0.0 | -2.0 | 3.8 |
| Related to relationship with partner | 2.2 | 11.3 | -5.9 |
| Related to relationship with children | -0.5 | -2.2 | 5.4 |
| Related to relationship with parents | 0.1 | 0.0 | -0.7 |
| Related to relationship with relatives | 0.0 | -0.5 | -0.3 |
| Related to free time | 0.0 | 0.8 | 2.5 |
| Related to finances | -2.6 | 2.0 | 28.8 |
| Related to own health | -1.6 | -9.4 | 4.2 |
| Related to school/study | 0.6 | 5.2 | -3.4 |
| Related to faith, church or religion | 0.1 | 0.7 | -2.2 |
| OR: odds ratio; CI: confidence interval; SEP: socioeconomic position; MetS: metabolic syndrome; LDI: Long-term Difficulties Inventory; ^a^ Long-term difficulties during total follow-up time measured with the LDI; analyses controlled for years of education, equivalized household income, occupational prestige, age and sex at T1, and time between T1 and T4; LDI domains were controlled for work status, partner status, children status, parent status, school/study status and religion status where applicable; *P<0.01. | | | |

**Supplementary Table 10. Multivariable logistic and linear regression analysis of direct associations between socioeconomic position, chronic stress^a^, and metabolic syndrome development among participants with more than 30% missings on variables (n=85,957).**

|  | **Education** | **Occupational prestige** | **Income** |
| --- | --- | --- | --- |
|  | **OR (99% CI)** | **OR (99% CI)** | **OR (99% CI)** |
| **Path 1. SEP and MetS development** | 0.92 (0.90, 0.94)* | 0.94 (0.91, 0.97)* | 1.00 (0.99, 1.00) |
| **Path 2. SEP and chronic stress^a^** |  |  |  |
| Sum score (beta) | 0.23 (0.20, 0.27)* | 0.16 (0.10, 0.21)* | -0.09 (-0.10, -0.08)* |
| Related to home and living |  |  |  |
| Slightly stressful | 1.10 (1.08, 1.11)* | 1.02 (1.00, 1.04) | 0.98 (0.98, 0.99)* |
| Very stressful | 1.16 (1.11, 1.21)* | 1.00 (0.93, 1.07) | 0.94 (0.93, 0.96)* |
| At or with work |  |  |  |
| Slightly stressful | 1.13 (1.12, 1.15)* | 1.09 (1.06, 1.11)* | 1.01 (1.00, 1.01)* |
| Very stressful | 1.23 (1.21, 1.26)* | 1.17 (1.13, 1.21)* | 1.02 (1.01, 1.02)* |
| Related to relationship with friends or acquaintances |  |  |  |
| Slightly stressful | 1.00 (0.99, 1.01) | 1.00 (0.98, 1.03) | 0.98 (0.97, 0.98)* |
| Very stressful | 0.99 (0.94, 1.05) | 0.97 (0.89, 1.06) | 0.93 (0.91, 0.96)* |
| Related to relationship with partner |  |  |  |
| Slightly stressful | 1.06 (1.04, 1.08)* | 1.03 (1.01, 1.06)* | 0.98 (0.98, 0.99)* |
| Very stressful | 1.12 (1.08, 1.15)* | 1.04 (1.00, 1.09)* | 0.96 (0.95, 0.97)* |
| Related to relationship with children |  |  |  |
| Slightly stressful | 1.05 (1.04, 1.06)* | 1.02 (1.00, 1.05)* | 0.96 (0.95, 0.96)* |
| Very stressful | 1.05 (1.01, 1.09)* | 1.09 (1.03, 1.15)* | 0.91 (0.89, 0.92)* |
| Related to relationship with parents |  |  |  |
| Slightly stressful | 1.01 (0.99, 1.03) | 1.00 (0.97, 1.03) | 0.99 (0.98, 0.99)* |
| Very stressful | 1.02 (0.98, 1.06) | 0.96 (0.90, 1.03) | 0.97 (0.95, 0.98)* |
| Related to relationship with relatives |  |  |  |
| Slightly stressful | 1.01 (1.00, 1.02) | 0.98 (0.96, 1.00) | 0.99 (0.99, 0.99)* |
| Very stressful | 1.00 (0.96, 1.04) | 0.97 (0.91, 1.04) | 0.97 (0.95, 0.99)* |
| Related to free time |  |  |  |
| Slightly stressful | 1.07 (1.06, 1.08)* | 1.05 (1.03, 1.07)* | 0.99 (0.99, 1.00)* |
| Very stressful | 1.13 (1.10, 1.16)* | 1.10 (1.05, 1.15)* | 0.97 (0.96, 0.98)* |
| Related to finances |  |  |  |
| Slightly stressful | 1.04 (1.03, 1.06)* | 1.00 (0.97, 1.02) | 0.93 (0.93, 0.94)* |
| Very stressful | 1.04 (1.00, 1.08) | 1.01 (0.95, 1.08) | 0.87 (0.86, 0.89)* |
| Related to own health |  |  |  |
| Slightly stressful | 1.02 (1.00, 1.03)* | 1.02 (1.00, 1.04)* | 0.99 (0.99, 1.00)* |
| Very stressful | 1.00 (0.97, 1.03) | 1.05 (1.00, 1.10)* | 0.97 (0.96, 0.98)* |
| Related to school/study |  |  |  |
| Slightly stressful | 1.06 (1.04, 1.09)* | 1.10 (1.06, 1.14)* | 0.99 (0.98, 1.00)* |
| Very stressful | 1.05 (0.98, 1.12) | 1.11 (1.00, 1.24) | 0.97 (0.94, 0.99)* |
| Related to faith, church or religion |  |  |  |
| Slightly stressful | 1.01 (0.99, 1.04) | 1.02 (0.99, 1.07) | 0.97 (0.95, 0.97)* |
| Very stressful | 1.17 (0.99, 1.39) | 0.86 (0.64, 1.15) | 0.95 (0.89, 1.01) |
| **Path 3. Chronic stress^a^ and MetS development** |  |  |  |
| Sum score | 1.02 (1.01, 1.02)* | 1.02 (1.01, 1.02)* | 1.02 (1.01, 1.02)* |
| Related to home and living |  |  |  |
| Slightly stressful | 0.89 (0.79, 0.99)* | 0.89 (0.79, 0.99)* | 0.89 (0.79, 0.99)* |
| Very stressful | 0.86 (0.64, 1.15) | 0.86 (0.64, 1.15) | 0.86 (0.64, 1.15) |
| At or with work |  |  |  |
| Slightly stressful | 1.00 (0.91, 1.09) | 1.00 (0.91, 1.09) | 1.00 (0.91, 1.09) |
| Very stressful | 1.06 (0.91, 1.24) | 1.06 (0.91, 1.24) | 1.06 (0.91, 1.24) |
| Related to relationship with friends or acquaintances |  |  |  |
| Slightly stressful | 1.07 (0.96, 1.18) | 1.07 (0.96, 1.18) | 1.07 (0.96, 1.18) |
| Very stressful | 1.19 (0.80, 1.77) | 1.19 (0.80, 1.77) | 1.19 (0.80, 1.77) |
| Related to relationship with partner |  |  |  |
| Slightly stressful | 0.83 (0.76, 0.92)* | 0.83 (0.76, 0.92)* | 0.83 (0.76, 0.92)* |
| Very stressful | 0.74 (0.60, 0.92)* | 0.74 (0.60, 0.92)* | 0.74 (0.60, 0.92)* |
| Related to relationship with children |  |  |  |
| Slightly stressful | 1.04 (0.94, 1.15) | 1.04 (0.94, 1.15) | 1.04 (0.94, 1.15) |
| Very stressful | 1.07 (0.84, 1.37) | 1.07 (0.84, 1.37) | 1.07 (0.84, 1.37) |
| Related to relationship with parents |  |  |  |
| Slightly stressful | 1.02 (0.91, 1.15) | 1.02 (0.91, 1.15) | 1.02 (0.91, 1.15) |
| Very stressful | 1.19 (0.90, 1.56) | 1.19 (0.90, 1.56) | 1.19 (0.90, 1.56) |
| Related to relationship with relatives |  |  |  |
| Slightly stressful | 1.04 (0.94, 1.15) | 1.04 (0.94, 1.15) | 1.04 (0.94, 1.15) |
| Very stressful | 1.03 (0.73, 1.45) | 1.03 (0.73, 1.45) | 1.03 (0.73, 1.45) |
| Related to free time |  |  |  |
| Slightly stressful | 1.02 (0.93, 1.11) | 1.02 (0.93, 1.11) | 1.02 (0.93, 1.11) |
| Very stressful | 1.04 (0.83, 1.30) | 1.04 (0.83, 1.30) | 1.04 (0.83, 1.30) |
| Related to finances |  |  |  |
| Slightly stressful | 1.27 (1.16, 1.39)* | 1.27 (1.16, 1.39)* | 1.27 (1.16, 1.39)* |
| Very stressful | 1.46 (1.15, 1.86)* | 1.46 (1.15, 1.86)* | 1.46 (1.15, 1.86)* |
| Related to own health |  |  |  |
| Slightly stressful | 1.27 (1.16, 1.39)* | 1.27 (1.16, 1.39)* | 1.27 (1.16, 1.39)* |
| Very stressful | 1.65 (1.36, 1.99)* | 1.65 (1.36, 1.99)* | 1.65 (1.36, 1.99)* |
| Related to school/study |  |  |  |
| Slightly stressful | 0.90 (0.78, 1.04) | 0.90 (0.78, 1.04) | 0.90 (0.78, 1.04) |
| Very stressful | 0.79 (0.49, 1.28) | 0.79 (0.49, 1.28) | 0.79 (0.49, 1.28) |
| Related to faith, church or religion |  |  |  |
| Slightly stressful | 0.98 (0.82, 1.18) | 0.98 (0.82, 1.18) | 0.98 (0.82, 1.18) |
| Very stressful | 1.11 (0.56, 2.20) | 1.11 (0.56, 2.20) | 1.11 (0.56, 2.20) |
| **Path 4. SEP and MetS development^b^** |  |  |  |
| Sum score | 0.92 (0.90, 0.93)* | 0.94 (0.91, 0.97)* | 1.00 (0.99, 1.00) |
| Related to home and living | 0.92 (0.90, 0.94)* | 0.94 (0.91, 0.97)* | 1.00 (0.99, 1.00) |
| At or with work | 0.92 (0.90, 0.93)* | 0.94 (0.91, 0.97)* | 1.00 (0.99, 1.00) |
| Related to relationship with friends or acquaintances | 0.92 (0.90, 0.94)* | 0.94 (0.91, 0.97)* | 1.00 (0.99, 1.00) |
| Related to relationship with partner | 0.92 (0.90, 0.94)* | 0.94 (0.91, 0.97)* | 1.00 (0.99, 1.00) |
| Related to relationship with children | 0.92 (0.91, 0.94)* | 0.94 (0.91, 0.97)* | 1.00 (0.99, 1.00) |
| Related to relationship with parents | 0.92 (0.90, 0.94)* | 0.94 (0.91, 0.97)* | 1.00 (0.99, 1.00) |
| Related to relationship with relatives | 0.92 (0.90, 0.94)* | 0.94 (0.92, 0.97)* | 1.00 (0.99, 1.00) |
| Related to free time | 0.92 (0.90, 0.94)* | 0.94 (0.91, 0.97)* | 1.00 (0.99, 1.00) |
| Related to finances | 0.92 (0.90, 0.93)* | 0.94 (0.91, 0.97)* | 1.00 (0.99, 1.01) |
| Related to own health | 0.92 (0.90, 0.94)* | 0.94 (0.91, 0.97)* | 1.00 (0.99, 1.00) |
| Related to school/study | 0.92 (0.90, 0.94)* | 0.94 (0.92, 0.97)* | 0.99 (0.99, 1.00) |
| Related to faith, church or religion | 0.92 (0.91, 0.94)* | 0.94 (0.91, 0.97)* | 0.99 (0.99, 1.00) |
| OR: odds ratio; CI: confidence interval; SEP: socioeconomic position; MetS: metabolic syndrome; LDI: Long-term Difficulties Inventory; ^a^ Long-term difficulties during total follow-up time measured with the LDI, LDI categories consist of the sum score of the LDI from questionnaires T2, T3 and T4, ‘not stressful’ indicates sum score 0, ‘slightly stressful’ sum score 1-3, ‘very stressful’ sum score 4-6; ^b^ Direct associations between SEP measures and MetS development controlled for specific LDI domain; analyses controlled for years of education, equivalized household income, occupational prestige, age and sex at T1, and time between T1 and T4; reference category for the LDI domains was ‘not stressful’; LDI domains were controlled for work status, partner status, children status, parent status, school/study status and religion status where applicable; *P<0.01. | | | |

**Supplementary Table 11. Multivariable mediation analysis of chronic stress^a^ in associations between socioeconomic position and metabolic syndrome development, using the Karlson-Holm-Breen method among participants with more than 30% missings on variables (n=85,957).**

|  | **Education** | **Occupational prestige** | **Income** |
| --- | --- | --- | --- |
|  | **OR (99% CI)** | **OR (99% CI)** | **OR (99% CI)** |
| **Total association** | 0.92 (0.91, 0.94)* | 0.94 (0.91, 0.97)* | 0.99 (0.99, 1.00) |
| **Direct association** | 0.92 (0.90, 0.94)* | 0.94 (0.91, 0.97)* | 1.00 (0.99, 1.00) |
| **Indirect association** | 1.00 (1.00, 1.01)* | 1.00 (1.00, 1.00)* | 1.00 (1.00, 1.00) |
|  |  |  |  |
|  | **Percentage** | **Percentage** | **Percentage** |
| **Mediating effect** |  |  |  |
| Sum score | -5.8 | -5.1 | 37.5 |
|  |  |  |  |
| **Mediating effects per domain of life** |  |  |  |
| Related to home and living | 2.4 | 0.8 | -11.0 |
| At or with work | -0.6 | -0.8 | -1.0 |
| Related to relationship with friends or acquaintances | -0.1 | -0.1 | 7.3 |
| Related to relationship with partner | 3.3 | 2.5 | -19.6 |
| Related to relationship with children | -0.4 | -0.4 | 7.9 |
| Related to relationship with parents | -0.2 | 0.1 | 2.6 |
| Related to relationship with relatives | -0.1 | 0.2 | 1.7 |
| Related to free time | -0.3 | -0.4 | 0.9 |
| Related to finances | -2.4 | 0.3 | 69.6 |
| Related to own health | -1.6 | -3.3 | 16.3 |
| Related to school/study | 0.3 | 0.8 | -3.0 |
| Related to faith, church or religion | 0.0 | 0.1 | -0.2 |
| OR: odds ratio; CI: confidence interval; SEP: socioeconomic position; MetS: metabolic syndrome; LDI: Long-term Difficulties Inventory; ^a^ Long-term difficulties during total follow-up time measured with the LDI; analyses controlled for years of education, equivalized household income, occupational prestige, age and sex at T1, and time between T1 and T4; LDI domains were controlled for work status, partner status, children status, parent status, school/study status and religion status where applicable; *P<0.01. | | | |

**Supplementary Table 12. Multivariable logistic and linear regression analysis of direct associations between socioeconomic position, chronic stress^a^, and metabolic syndrome development among complete cases (n=41,455).**

|  | **Education** | **Occupational prestige** | **Income** |
| --- | --- | --- | --- |
|  | **OR (99% CI)** | **OR (99% CI)** | **OR (99% CI)** |
| **Path 1. SEP and MetS development** | 0.92 (0.90, 0.95)* | 0.95 (0.90, 0.99)* | 0.99 (0.98, 1.00) |
| **Path 2. SEP and chronic stress^a^** |  |  |  |
| Sum score (beta) | 0.20 (0.17, 0.24)* | 0.16 (0.10, 0.22)* | -0.09 (-0.10, -0.08)* |
| Related to home and living |  |  |  |
| Slightly stressful | 1.10 (1.08, 1.13)* | 1.01 (0.98, 1.05) | 0.98 (0.97, 0.99)* |
| Very stressful | 1.15 (1.08, 1.22)* | 0.94 (0.85, 1.03) | 0.95 (0.93, 0.97)* |
| At or with work |  |  |  |
| Slightly stressful | 1.13 (1.11, 1.15)* | 1.09 (1.06, 1.12)* | 1.01 (1.00, 1.01)* |
| Very stressful | 1.18 (1.15, 1.22)* | 1.16 (1.11, 1.21)* | 1.02 (1.01, 1.03)* |
| Related to relationship with friends or acquaintances |  |  |  |
| Slightly stressful | 0.99 (0.98, 1.01) | 1.00 (0.97, 1.03) | 0.98 (0.97, 0.98)* |
| Very stressful | 0.96 (0.89, 1.05) | 0.98 (0.85, 1.12) | 0.94 (0.92, 0.97)* |
| Related to relationship with partner |  |  |  |
| Slightly stressful | 1.05 (1.03, 1.07)* | 1.03 (1.01, 1.06)* | 0.98 (0.98, 0.99)* |
| Very stressful | 1.10 (1.06, 1.14)* | 1.05 (0.98, 1.11) | 0.97 (0.95, 0.98)* |
| Related to relationship with children |  |  |  |
| Slightly stressful | 1.05 (1.03, 1.06)* | 1.02 (0.99, 1.05) | 0.96 (0.95, 0.97)* |
| Very stressful | 1.02 (0.97, 1.08) | 1.11 (1.02, 1.21)* | 0.91 (0.89, 0.93)* |
| Related to relationship with parents |  |  |  |
| Slightly stressful | 1.01 (0.99, 1.03) | 1.00 (0.97, 1.03) | 0.99 (0.98, 0.99)* |
| Very stressful | 1.02 (0.97, 1.09) | 0.95 (0.87, 1.05) | 0.97 (0.95, 0.99)* |
| Related to relationship with relatives |  |  |  |
| Slightly stressful | 1.00 (0.99, 1.02) | 0.97 (0.95, 1.00)* | 0.99 (0.98, 1.00)* |
| Very stressful | 0.99 (0.93, 1.05) | 0.97 (0.88, 1.08) | 0.99 (0.97, 1.02) |
| Related to free time |  |  |  |
| Slightly stressful | 1.07 (1.05, 1.08)* | 1.05 (1.02, 1.07)* | 1.00 (0.99, 1.00) |
| Very stressful | 1.11 (1.06, 1.15)* | 1.13 (1.06, 1.21)* | 0.97 (0.96, 0.99)* |
| Related to finances |  |  |  |
| Slightly stressful | 1.05 (1.03, 1.07)* | 0.99 (0.96, 1.02) | 0.93 (0.92, 0.93)* |
| Very stressful | 1.02 (0.97, 1.08) | 1.03 (0.94, 1.12) | 0.88 (0.86, 0.90)* |
| Related to own health |  |  |  |
| Slightly stressful | 1.02 (1.00, 1.03)* | 1.02 (1.00, 1.05) | 0.99 (0.99, 1.00)* |
| Very stressful | 1.01 (0.97, 1.05) | 1.03 (0.97, 1.10) | 0.98 (0.97, 1.00)* |
| Related to school/study |  |  |  |
| Slightly stressful | 1.05 (1.02, 1.08)* | 1.10 (1.05, 1.14)* | 0.99 (0.98, 1.00)* |
| Very stressful | 1.01 (0.94, 1.10) | 1.11 (0.99, 1.25) | 0.97 (0.95, 1.00) |
| Related to faith, church or religion |  |  |  |
| Slightly stressful | 1.00 (0.97, 1.04) | 1.03 (0.98, 1.09) | 0.97 (0.95, 0.98)* |
| Very stressful | 1.11 (0.91, 1.37) | 0.91 (0.65, 1.28) | 0.94 (0.87, 1.02) |
| **Path 3. Chronic stress^a^ and MetS development** |  |  |  |
| Sum score | 1.02 (1.01, 1.03)* | 1.02 (1.01, 1.03)* | 1.02 (1.01, 1.03)* |
| Related to home and living |  |  |  |
| Slightly stressful | 0.86 (0.75, 1.00) | 0.86 (0.75, 1.00) | 0.86 (0.75, 1.00) |
| Very stressful | 0.93 (0.61, 1.43) | 0.93 (0.61, 1.43) | 0.93 (0.61, 1.43) |
| At or with work |  |  |  |
| Slightly stressful | 1.04 (0.92, 1.17) | 1.04 (0.92, 1.17) | 1.04 (0.92, 1.17) |
| Very stressful | 1.07 (0.87, 1.31) | 1.07 (0.87, 1.31) | 1.07 (0.87, 1.31) |
| Related to relationship with friends or acquaintances |  |  |  |
| Slightly stressful | 1.04 (0.90, 1.19) | 1.04 (0.90, 1.19) | 1.04 (0.90, 1.19) |
| Very stressful | 1.59 (0.94, 2.70) | 1.59 (0.94, 2.70) | 1.59 (0.94, 2.70) |
| Related to relationship with partner |  |  |  |
| Slightly stressful | 0.81 (0.71, 0.92)* | 0.81 (0.71, 0.92)* | 0.81 (0.71, 0.92)* |
| Very stressful | 0.79 (0.59, 1.06) | 0.79 (0.59, 1.06) | 0.79 (0.59, 1.06) |
| Related to relationship with children |  |  |  |
| Slightly stressful | 1.05 (0.93, 1.20) | 1.05 (0.93, 1.20) | 1.05 (0.93, 1.20) |
| Very stressful | 0.95 (0.66, 1.39) | 0.95 (0.66, 1.39) | 0.95 (0.66, 1.39) |
| Related to relationship with parents |  |  |  |
| Slightly stressful | 0.93 (0.80, 1.09) | 0.93 (0.80, 1.09) | 0.93 (0.80, 1.09) |
| Very stressful | 1.07 (0.71, 1.62) | 1.07 (0.71, 1.62) | 1.07 (0.71, 1.62) |
| Related to relationship with relatives |  |  |  |
| Slightly stressful | 1.10 (0.97, 1.25) | 1.10 (0.97, 1.25) | 1.10 (0.97, 1.25) |
| Very stressful | 1.23 (0.81, 1.88) | 1.23 (0.81, 1.88) | 1.23 (0.81, 1.88) |
| Related to free time |  |  |  |
| Slightly stressful | 1.00 (0.89, 1.12) | 1.00 (0.89, 1.12) | 1.00 (0.89, 1.12) |
| Very stressful | 0.98 (0.72, 1.33) | 0.98 (0.72, 1.33) | 0.98 (0.72, 1.33) |
| Related to finances |  |  |  |
| Slightly stressful | 1.34 (1.17, 1.54)* | 1.34 (1.17, 1.54)* | 1.34 (1.17, 1.54)* |
| Very stressful | 1.47 (1.03, 2.11)* | 1.47 (1.03, 2.11)* | 1.47 (1.03, 2.11)* |
| Related to own health |  |  |  |
| Slightly stressful | 1.30 (1.17, 1.46)* | 1.30 (1.17, 1.46)* | 1.30 (1.17, 1.46)* |
| Very stressful | 1.78 (1.39, 2.28)* | 1.78 (1.39, 2.28)* | 1.78 (1.39, 2.28)* |
| Related to school/study |  |  |  |
| Slightly stressful | 0.98 (0.81, 1.19) | 0.98 (0.81, 1.19) | 0.98 (0.81, 1.19) |
| Very stressful | 0.93 (0.47, 1.86) | 0.93 (0.47, 1.86) | 0.93 (0.47, 1.86) |
| Related to faith, church or religion |  |  |  |
| Slightly stressful | 1.00 (0.79, 1.28) | 1.00 (0.79, 1.28) | 1.00 (0.79, 1.28) |
| Very stressful | 1.27 (0.37, 4.38) | 1.27 (0.37, 4.38) | 1.27 (0.37, 4.38) |
| **Path 4. SEP and MetS development^b^** |  |  |  |
| Sum score | 0.92 (0.90, 0.94)* | 0.94 (0.90, 0.99)* | 1.00 (0.99, 1.01) |
| Related to home and living | 0.92 (0.90, 0.95)* | 0.95 (0.90, 0.99)* | 0.99 (0.98, 1.00) |
| At or with work | 0.92 (0.90, 0.94)* | 0.94 (0.90, 0.99)* | 0.99 (0.98, 1.00) |
| Related to relationship with friends or acquaintances | 0.92 (0.90, 0.95)* | 0.95 (0.90, 0.99)* | 0.99 (0.98, 1.00) |
| Related to relationship with partner | 0.92 (0.90, 0.95)* | 0.95 (0.90, 0.99)* | 0.99 (0.98, 1.00) |
| Related to relationship with children | 0.92 (0.90, 0.95)* | 0.95 (0.90, 0.99)* | 0.99 (0.98, 1.00) |
| Related to relationship with parents | 0.92 (0.90, 0.95)* | 0.95 (0.90, 0.99)* | 0.99 (0.98, 1.00) |
| Related to relationship with relatives | 0.92 (0.90, 0.95)* | 0.95 (0.90, 0.99)* | 0.99 (0.98, 1.00) |
| Related to free time | 0.92 (0.90, 0.95)* | 0.94 (0.90, 0.99)* | 0.99 (0.98, 1.00) |
| Related to finances | 0.92 (0.90, 0.95)* | 0.95 (0.90, 0.99)* | 1.00 (0.99, 1.01) |
| Related to own health | 0.92 (0.90, 0.95)* | 0.94 (0.90, 0.99)* | 0.99 (0.98, 1.00) |
| Related to school/study | 0.92 (0.90, 0.95)* | 0.95 (0.90, 0.99)* | 0.99 (0.98, 1.00) |
| Related to faith, church or religion | 0.92 (0.90, 0.95)* | 0.95 (0.90, 0.99)* | 0.99 (0.98, 1.00) |

OR: odds ratio; CI: confidence interval; SEP: socioeconomic position; MetS: metabolic syndrome; LDI: Long-term Difficulties Inventory; ^a^ Long-term difficulties during total follow-up time measured with the LDI, LDI categories consist of the sum score of the LDI from questionnaires T2, T3 and T4, ‘not stressful’ indicates sum score 0, ‘slightly stressful’ sum score 1-3, ‘very stressful’ sum score 4-6; ^b^ Direct associations between SEP measures and MetS development controlled for specific LDI domain; analyses controlled for years of education, equivalized household income, occupational prestige, age and sex at T1, and time between T1 and T4; reference category for the LDI domains was ‘not stressful’; LDI domains were controlled for work status, partner status, children status, parent status, school/study status and religion status where applicable; *P<0.01.

**Supplementary Table 13. Multivariable mediation analysis of chronic stress^a^ in associations between socioeconomic position and metabolic syndrome development, using the Karlson-Holm-Breen method among complete cases (n=41,455).**

|  | **Education** | **Occupational prestige** | **Income** |
| --- | --- | --- | --- |
|  | **OR (99% CI)** | **OR (99% CI)** | **OR (99% CI)** |
| **Total association** | 0.92 (0.91, 0.94)* | 0.94 (0.91, 0.98)* | 0.99 (0.99, 1.00) |
| **Direct association** | 0.92 (0.90, 0.94)* | 0.94 (0.91, 0.97)* | 1.00 (0.99, 1.00) |
| **Indirect association** | 1.00 (1.00, 1.01)* | 1.00 (1.00, 1.00)* | 1.00 (1.00, 1.00)* |
|  |  |  |  |
|  | **Percentage** | **Percentage** | **Percentage** |
| **Mediating effect** |  |  |  |
| Sum score | -5.2 | -5.7 | 29.4 |
|  |  |  |  |
| **Mediating effects per domain of life** |  |  |  |
| Related to home and living | 2.6 | 0.9 | -9.9 |
| At or with work | -1.3 | -1.6 | -1.5 |
| Related to relationship with friends or acquaintances | 0.1 | 0.1 | 5.1 |
| Related to relationship with partner | 2.9 | 2.8 | -15.9 |
| Related to relationship with children | -0.4 | -0.1 | 5.0 |
| Related to relationship with parents | 0.1 | 0.2 | -1.5 |
| Related to relationship with relatives | 0.0 | 1.0 | 3.1 |
| Related to free time | 0.1 | 0.1 | -0.3 |
| Related to finances | -2.5 | 0.4 | 66.3 |
| Related to own health | -1.5 | -3.1 | 13.8 |
| Related to school/study | 0.0 | 0.3 | -0.9 |
| Related to faith, church or religion | 0.0 | 0.0 | 0.3 |
| OR: odds ratio; CI: confidence interval; SEP: socioeconomic position; MetS: metabolic syndrome; LDI: Long-term Difficulties Inventory; ^a^ Long-term difficulties during total follow-up time measured with the LDI; analyses controlled for years of education, equivalized household income, occupational prestige, age and sex at T1, and time between T1 and T4; LDI domains were controlled for work status, partner status, children status, parent status, school/study status and religion status where applicable; *P<0.01. | | | |

**Supplementary Table 14. Interaction coefficients of sex*SEP in the multivariable logistic regression analyses between socioeconomic position measures and metabolic syndrome development in the study population (n=53,216).**

| **Socioeconomic position measure** | **OR (99% CI)** |
| --- | --- |
| Female*Education | 0.97 (0.93, 1.01) |
| Female*Income | 1.00 (0.98, 1.02) |
| Female*Occupational prestige | 0.94 (0.88, 1.01) |

OR: odds ratio; CI: confidence interval; all analyses were controlled for years of education, equivalized household income, occupational prestige, age and sex at T1 and time between T1 and T4; reference category was ‘male*socioeconomic position measure’; *P<0.01.
